# Transcriptome analyses of immune tissues from three Japanese frogs (genus *Rana*) reveals their utility in characterizing major histocompatibility complex class II

**DOI:** 10.1186/s12864-017-4404-0

**Published:** 2017-12-28

**Authors:** Quintin Lau, Takeshi Igawa, Ryuhei Minei, Tiffany A. Kosch, Yoko Satta

**Affiliations:** 1 Department of Evolutionary Studies of Biosystems, Sokendai, The Graduate University for Advanced Studies, Kamiyamaguchi 1560-35, Hayama, Kanagawa 240-0193 Japan; 2 Amphibian Research Center, Hiroshima University, 1-3-1, Higashi-Hiroshima, Hiroshima, 739-8526 Japan; 3 Department of Bioscience, Nagahama Institute of Bio-Science and Technology, Tamura-cho 1266, Nagahama, Shiga 526-0829 Japan; 4 One Health Research Group, College of Public Health, Medical and Veterinary Sciences, James Cook University, Townsville, QLD 4811 Australia

**Keywords:** Ranidae, Chytridiomycosis, MHC supertypes, RNA-Seq, *Rana japonica*, *Rana ornativentris*, *Rana tagoi*, Antimicrobial peptides

## Abstract

**Background:**

In Japan and East Asia, endemic frogs appear to be tolerant or not susceptible to chytridiomycosis, a deadly amphibian disease caused by the chytrid fungus *Batrachochytridium dendrobatidis* (Bd). Japanese frogs may have evolved mechanisms of immune resistance to pathogens such as Bd. This study characterizes immune genes expressed in various tissues of healthy Japanese *Rana* frogs.

**Results:**

We generated transcriptome data sets of skin, spleen and blood from three adult Japanese Ranidae frogs (Japanese brown frog *Rana japonica*, the montane brown frog *Rana ornativentris*, and Tago’s brown frog *Rana tagoi tagoi*) as well as whole body of *R. japonica* and *R. ornativentris* tadpoles. From this, we identified tissue- and stage-specific differentially expressed genes; in particular, the spleen was most enriched for immune-related genes. A specific immune gene, major histocompatibility complex class IIB (MHC-IIB), was further characterized due to its role in pathogen recognition. We identified a total of 33 MHC-IIB variants from the three focal species (*n* = 7 individuals each), which displayed evolutionary signatures related to increased MHC variation, including balancing selection. Our supertyping analyses of MHC-IIB variants from Japanese frogs and previously studied frog species identified potential physiochemical properties of MHC-II that may be important for recognizing and binding chytrid-related antigens.

**Conclusions:**

This is one of the first studies to generate transcriptomic resources for Japanese frogs, and contributes to further understanding the immunogenetic factors associated with resistance to infectious diseases in amphibians such as chytridiomycosis. Notably, MHC-IIB supertyping analyses identified unique functional properties of specific MHC-IIB alleles that may partially contribute to Bd resistance, and such properties provide a springboard for future experimental validation.

**Electronic supplementary material:**

The online version of this article (10.1186/s12864-017-4404-0) contains supplementary material, which is available to authorized users.

## Background

Changes in the host-pathogen-environment interactions can lead to differential susceptibility to infectious disease. Anuran amphibians experience marked changes in physiology and environment due to their biphasic life cycle which includes pre-metamorphic tadpoles that inhabit fully aqueous environments, and post-metamorphic adults that inhabit aqueous and/or terrestrial environments. Anurans undergo complete physiological reorganization at metamorphosis, including the immune system. Some ontological differences in immunity between tadpole and adult frogs include turnover of lymphocytes at metamorphosis, differences in antibody responses and expression of major histocompatibility complex (MHC) antigens (reviewed in [1]). Influence of life stage on immunity is supported by evidence that pathogens (e.g. protists, ranaviruses, fungi) differentially affect either tadpole or adult frog stages [2–4]. In addition, variation in habitat can impact the interaction of pathogens and host immune response [5], whereby pathogen diversity could differ between the aqueous environment of tadpoles and the terrestrial /aquatic lifestyle of adult frogs.

Chytridiomycosis is a devastating disease in amphibians caused by the chytrid fungus *Batrachochytridium dendrobatidis* (Bd). Bd infection results in epidermal disruption and pathophysiological changes sometimes leading to mortality [6], and has been attributed to the decline of amphibian populations worldwide [7–10]. Although variable Bd prevalence in Korea [11] and Japan [12] has been found, there is a lack of Bd related declines reported in East Asia as well as no evidence of Bd susceptibility in endemic East Asian frogs following experimental infection. There is an ongoing theory that Bd is endemic to Asia [11, 13], so East Asian and Japanese frogs may have had a lengthy co-evolution with the pathogenic fungi and acquired immune tolerance. Therefore, study of immune genes from Japanese amphibian species is important for further understanding amphibian-chytridiomycosis dynamics.

Major histocompatibility complex (MHC) are one of the most polymorphic gene families in vertebrates [14]. They code for membrane-bound glycoproteins that recognize, bind and present antigenic peptides to T lymphocytes, and thus are essential for adaptive immunity in jawed vertebrates. There are two major classes of MHC molecules: MHC class I (MHC-I) predominantly recognize and present endogenous antigenic peptides such as from viruses, while MHC class II (MHC-II) detect and present exogenous-derived peptides such as from bacteria and fungi [15]; because of this, studies of MHC genetics in East Asian frogs in the context of Bd fungus have largely focused on characterization of MHC-II genes [16–18]. MHC-II proteins are comprised of non-covalently associated alpha (α or MHC-IIA) and beta (β or MHC-IIB) chains, each with two extracellular domains (α1 and α2, and β1 and β2, respectively). The peptide binding region of the β1 chain has the highest variation, and this diversity governs the repertoire of antigenic determinants to which the host individual can respond [19].

MHC diversity is maintained predominantly by pathogen-mediated balancing selection in an evolutionary time scale [20], and several vertebrate studies have found associations between MHC genetic variation and infectious diseases (reviewed in [21]). In the case of amphibian-chytridiomycosis dynamics, MHC-IIB conformation was suggested to be associated with resistance to Bd [18]; this was based chiefly on amino acid properties at P9 binding pockets that were conserved between resistant Korean frog species and individuals of Australian alpine tree frogs (*Litoria verreauxii alpina*) that survived Bd infection. Other studies in non-Asian frog species have also identified associations between chytridiomycosis with MHC-IIB alleles or supertypes in wild and captive frogs [22, 23]; supertyping is a method for grouping alleles based on amino acid properties. While such studies have identified MHC class II-chytridiomycosis associations, resistance to disease is often elicited by polygenetic responses and this should be considered. Within Japanese frogs, immunogenetic studies have been limited to skin antimicrobial peptides (AMPs) and MHC class I from Ranidae species [24, 25]. With the increasing availability of next-generation sequencing technologies, the application of transcriptome analyses and genome-wide SNPs is now accessible to non-model organisms including frogs [26–28].

In Japan, brown frogs of true frog genus *Rana* are a common frog species distributed throughout the Japanese archipelago. Of these, three species, Japanese brown frog *Rana japonica*, the montane brown frog *Rana ornativentris*, and Tago’s brown frog *Rana tagoi tagoi,* are found on Honshu (Japanese mainland). Although they are distributed in neighboring areas, each species inhabits different habitats: grasslands from lowland to hillsides (*R. japonica*), lowland to montane area (*R. ornativentris*), and montane area close to torrents (*R. t. tagoi*). This can provide an intrinsic opportunity to compare the expression patterns of immune-related genes and validate selective traits that may be conserved across a variety of ecological and evolutionary backgrounds. The aim of this study is to perform transcriptome analyses of immune-related tissues from these three Japanese *Rana* frog species and compare expression of genes associated with immune function such as MHC and AMPs. We then focused on MHC-IIB, which may be important for chytridiomycosis resistance, for further analyses of expression and genetic characterization.

## Results

### Transcriptome data set and differential expression between tissues and life stages

We used our Illumina sequence results to assemble clean reads from each of the three Japanese *Rana* species into 303,238–646,586 transcripts with an average contig size of 561–650 bp (Table 1). Our BLAST search of all assembled transcripts against sequences of the Swissprot, human Ensembl, Protein family (Pfam), Kyoto Encyclopedia of Genes and Genomes (KEGG) Orthology (KO), and Gene Ontology (GO) databases indicated that among all assembled contigs, 16.61% to 19.45% were annotated using BLAST search in at least one database (Table 1).

**Table 1:** Summary statistics of Illumina sequencing, assembly and annotation of normalized transcriptomes from three Japanese Ranidae species, including AMP transcripts identified

Species	Samples (No. of immune-related enriched GO terms)	No. of assembled contigs	Average contig length (bp)	No. annotated contigs						AMP gene families identified
				Uniprot	Ensembl human	GO	Pfam	KO	Total	
*R. japonica*	blood (2) skin (6^a^) spleen (46^a^) s29 tadpole body (2)	312,172	650	54,984 (17.61%)	49,635 (15.90%)	52,377 (16.79%)	36,646 (11.74%)	42,592 (13.64%)	57,339 (18.37%)	bradykinin, brevinin^b^, histone, japonicin^b^, kassorin, kininogen, nigrosin, odorranain, pleurain, preprobrevinin^b^, preprochensinin, ranacyclin, ranatensin, temporin
*R. ornativentris*	blood (5) skin (7^a^) spleen (19^a^) s24 tadpole skin (3^a^) s29 tadpole body (3^a^)	646,586	561	101,666 (15.72%)	88,074 (13.62%)	97,143 (15.02%)	61,503 (9.51%)	77,264 (11.95%)	107,391 (16.61%)	andersonin, bombesin, bradykinin, brevinin, gaegurin, histone, japonicin, kininogen, nigroain, odorranain^b^, peptide DK25, pleurain^b^, preprobrevinin^b^, preprochensinin^b^, prepropalustrin, preproranatuerin, ranacyclin, ranatuerin^b^, tigerinin
*R. t. tagoi*	blood (6^a^) skin (3) spleen (38)	303,238	634	56,202 (18.54%)	49,321 (16.26%)	53,866 (17.76%)	34,432 (11.35%)	42,951 (14.16%)	58,980 (19.45%)	andersonin, bradykinin^b^, brevinin, daunchinain, esculentin, gaegurin, histone, japonicin, kininogen, lividin, preprobrevinin^b^, preprochensinin, prepropalustrin, ranatuerin, temporin^b^

Percentages of contigs annotated against each database are shown in parentheses. ^a^list of enriched GO terms includes GO:0019886: antigen processing and presentation of exogenous peptide antigen via MHC class II. ^b^indicates 2 transcripts were found within a particular AMP family

**Fig. 1:**
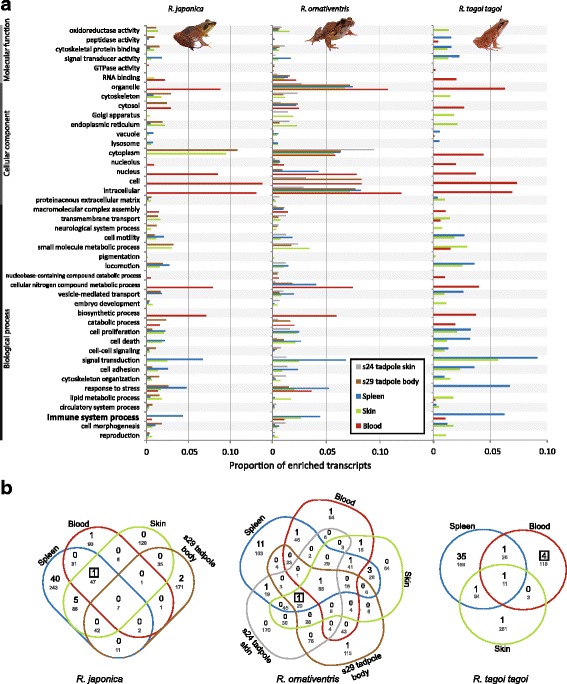
Transcriptome data was utilized to identify differentially expressed gene ontology (GO) categories between tissue types and life stages. **a** Summary of all enriched transcripts (proportion > 0.01) distributed into level two GO categories, run independently in each of the three focal frog species. Only GO categories enriched in a specific adult tissue (blood, skin, spleen) across all three species, or enriched in tadpoles compared to adult tissues in both *R. japonica* and *R. ornativentris*, are presented (full details in Additional file 1: Table S2). **b** Venn diagrams of full GO term enriched groups in blood, skin, blood (and tadpole) from the three species. Number of immune-related enriched GO terms are in black (summarized in Additional file 1: Tables S3 - S5), while total number of enriched GO terms belonging to the ‘biological process’ parent category are in grey. Position of term GO:0019886 (antigen processing and presentation of exogenous peptide antigen via MHC class II) is indicated by boxed number. Photo source: Q. Lau

Using the transcriptome data sets derived from RNA samples, we extracted 917–32,363 differentially expressed (DE) transcripts from pairwise comparisons within each of the three focal species. This corresponded to 531–7160 DE genes after encoding with Ensembl gene IDs and 8–74 GO-slim (GO level two) categories, with highest number of DE genes and GO categories in adult-tadpole pairwise comparisons (Additional file 1: Table S1). Several level two GO categories were enriched in specific adult tissues across all three frog species studied (Fig. 1a, Additional file 1: Table S2): for example, in all species spleen was enriched in ‘immune system process’, ‘cell death’ and ‘cell proliferation’, while blood was enriched for several terms within the ‘cellular component’ parent category (‘nucleus’, ‘cell’, ‘intracellular’), and skin was enriched for ‘small molecule metabolic process’ and ‘oxidoreductase activity’. In addition, several GO categories were enriched in tadpoles compared to adult tissues in both *R. japonica* and *R. ornativentris* (Fig. 1a, Additional file 1: Table S2).These included ‘cytoskeleton organization’, ‘cell-cell signaling’, and ‘cytoskeletal protein binding’.

### Differential expression of immune-related genes, including MHC and antimicrobial peptides (AMPs)

Further examination centering on the biological process parent term ‘immune system process’ confirmed that the spleen contained the most enriched immune-related GO terms in all three species, with 19 to 46 GO terms (Fig. 1b, Additional file 1: Tables S3 to S5). Nine of the GO terms were common in spleen of all three species: inflammatory response (GO:0006954) and its regulation and negative regulation (GO:0050727 and GO:0050728), leukocyte cell-cell adhesion (GO:0007159) and migration (GO:0050900), T cell differentiation (GO:0030217) and homeostasis (GO:0043029), T cell receptor signaling pathway (GO:0050852), and positive regulation of B cell differentiation (GO:0045579). In blood and skin samples of the focal species, two to seven immune-related GO terms were enriched; of these, only regulation of inflammatory response (GO:0050727) in the skin was enriched across all species. Among adult frogs, the MHC class II related GO term (GO:0019886) was enriched in various samples, including spleen and skin of *R. ornativentris* and *R. japonica*, and blood of *R. t. tagoi* (Fig. 1B, Additional file 1: Tables S3 - S5). Similarly, expression of MHC class II transcripts/variants was the highest in the spleen, with the exception of the *Rata*01 variant (highest expression in blood, Table 2). In addition, MHC class I normalized expression was higher in spleen and blood and lower in tadpoles (Table 2).

**Table 2:** Normalized expression of MHC class IIB variants as well as select MHC class I and AMP sequences identified from transcriptome data of three Japanese *Rana* species, represented by TMM (trimmed mean of log expression ratio) values

Species	Gene or variant	Blood TMM	Skin TMM	Spleen TMM	S24 tadpole skin TMM	S29 tadpole body TMM
*R. japonica*	MHCII-*Raja*01	0.7	5.4	169.3	–	0.0
	MHCII-*Raja*03	9.1	44.3	382.3	–	0.4
	MHC class I	139.5	27.2	142.7	–	6.3
	Brevinin-1Toa	0.0	0.7	0.0	–	26.4
	Histone-H2B	3.3	3.1	1.1	–	2.8
	Japonicin-1NPb	0.0	22.5	0.2	–	0.0
	Japonicin-2Ja	0.0	2573.5	1.9	–	0.3
	Preprobrevinin-1Ja	0.0	327.0	0.2		62.4
*R. ornativentris*	MHCII-*Raor*01	57.6	46.6	84.1	0.0	0.6
	MHC class I	64.8	11.5	26.5	0.0	0.0
	Andersonin-R	0.0	0.0	0.0	0.0	30.7
	Brevinin-1RTb	0.5	760.2	1.0	0.0	0.0
	Histone-H2B	1.9	1.7	2.0	6.5	9.9
	Japonicin-2Ja	0.0	0.0	0.0	1.2	0.0
	Preprobrevinin-1Ka	0.0	572.3	0.0	0.0	0.0
	Preproranatuerin-2 Oe	0.0	740.4	0.0	0.0	0.0
*R. t. tagoi*	MHCII-*Rata*01	138.8	14.3	37.2	–	–
	MHCII-*Rata*03	24.2	8.1	46.0	–	–
	MHC class I	256.8	12.5	65.1	–	–
	Andersonin-R	0.0	20.3	0.0	–	–
	Brevinin-1Toa	0.0	222.0	0.0	–	–
	Histone-H2B	0.1	9.2	0.7	–	–
	Japonicin-1NPb	0.0	325.4	0.3	–	–
	Preprobrevinin-1Ka	0.0	883.1	0.0	–	–
	Temporin-GN2	0.0	50.2	0.0	–	–

When comparing adult with tadpole samples, there were no overlapping immune-related GO terms enriched in either *R. japonica* or *R. ornativentris* tadpoles. In the *R. japonica* tadpole, two terms related to complement activation were enriched (GO:0030449 and GO:0006957). In the *R. ornativentris* tadpole, terms related to intracellular transport of virus (GO:0075733), and interestingly, antigen processing and presentation of exogenous peptide antigen via MHC class I and II (GO:0002479 and GO:0019886, respectively) were enriched. An in-depth look into the GO:0019886 term revealed that genes coding for the MHC class II protein were not upregulated in *R. ornativentris* tadpoles, but rather genes related to intracellular assembly and transport of the MHC class II protein (Additional file 1: Table S6). This was further validated by relatively low expression levels of MHC class II transcripts in tadpoles (Table 2).

While no specific GO terms related to antimicrobial peptides (AMPs) were enriched, we isolated several AMP transcripts from the three *Rana* species using tblastn search (17–24 AMP genes per species, Table 1). Seven AMP families were identified in all three species- bradykinin, histone, japonicin, kininogen, preprobrevinin, and preprochensinin. From normalized expression values of AMP genes (Table 2, Additional file 1: Table S7), highest expression was predominant in adult skin relative to all other samples including all 17 AMP genes of *R. t. tagoi.* Meanwhile, some AMP genes had variable expression: for example, histone was not highly expressed in the skin of all species, and some AMP genes had higher expression in tadpoles, like brevinin-1Toa and andersonin–R in *R. japonica* and *R. ornativentris*, respectively (Table 2).

### MHC class II sequence analyses

**Fig. 2:**
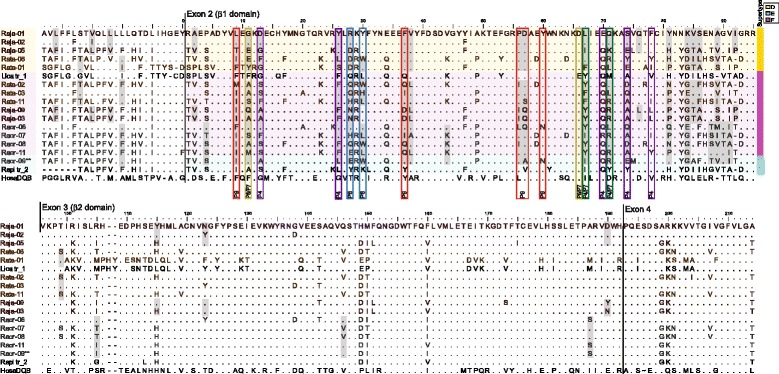
Amino acid alignment of selected MHC-IIB variants from three Japanese *Rana* species (*R. japonica- Raja*, *R. ornativentris- Raor,* and *R. t. tagoi- Rata*) spanning from exons 1 to 4. All β1 domain (exon 2) sequences from these species were allocated to one of three supertypes (ST-D, ST-E and ST-F), indicated on the right. Positive selected codon sites detected using omegaMap are shaded in grey. Amphibian peptide binding residues [18] are indicated by coloured boxes that represent pocket residues (P4, P6,P7 or P9). For supertyping analyses, the first three pocket residues were excluded due to absent sequence information in some other species (position 9, 11, and 13). Sequences were also included for human-*Hosa*DQB (Genbank accession M33907.1), and transcriptome sequences obtained from *R.pirica- Rapi* and *L. catesbeianus-Lica*. **The *Raor*-09 variant is identical at the amino acid level with *Rata*-04, *Raor*-01 and *Raor*-02 variants

**Fig. 3:**
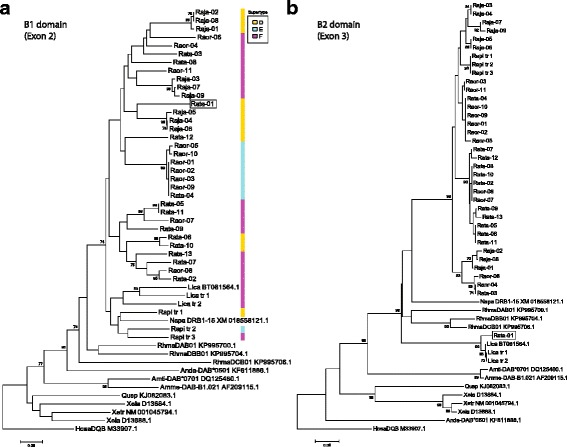
Phylogenetic relationships between MHC-IIB variants (amino acid sequences) identified in *R. japonica (Raja), R. ornativentris (Raor), and R. t. tagoi (Rata)* and other amphibians using neighbour-joining method and 1000 bootstrap replicates. Phylogenies were constructed based from (**a**) entire β1 domain encoded by exon 2, and (**b**) entire β2 domain encoded by exon 3. Nodes with bootstrap support >70% are indicated. Accession numbers from other species are indicated on labels, and ‘tr’ indicates sequences obtained from transcriptomic data of *R. pirica* (Mori, T., unpublished) and *L. catesbeianus* (DRA accession number SRP051787). The unique variant *Rata*-01, which clusters differently in each exon, is emphasised by a box. Supertypes allocated to β1 domain variants are indicated by coloured lines; other species used for supertype analyses are not included due to incomplete sequences

From molecular cloning we identified nine, 11, and 13 MHC-IIB sequences from *R. japonica, R. ornativentris* and *R. t. tagoi*, respectively (*n* = 7 individuals per species, accession numbers MF555153-MF555185). Amplification of multiple loci was only evident in *R. japonica*, where two to three sequences were identified per individual (Table 3). Seven of the nine *R. japonica* MHC-IIB variants were shared within the population studied. For the other two focal species, only *Raor*01, *Raor*02, and *Rata*01 variants were shared between individuals. However, identical nucleotide sequences were shared between a *R. ornativentris* and a *R. tagoi tagoi* individual (*Raor*09 and *Rata*04, Fig. 2), and these two variants were identical at the amino acid level with *Raor*01 and *Raor*02 found in other individuals. Interestingly, we also found that the *Rata*01 variant had high exon 3 sequence similarity to that of the American bullfrog (*L. catesbeianus*, Fig. 2); this divergent variant clusters phylogenetically with *L. catesbeianus* at β2 domain (exon 3) but not β1 domain (exon 2) sequences (Fig. 3, Additional file 1: figure S1). There was limited species-specific phylogenetic clustering in either β1 or β2 domain; some variants from different species clustered with strong bootstrap support, for example *Raor*07 with *Rata*05/11, and *Raor*08 with *Rata*02 (Fig. 3). The number of segregating sites as well as sequence divergence within individuals was highest in *R. t. tagoi*, but following exclusion of the divergent *Rata*01 variant that was found in a single individual, the parameters were comparable between the three focal species (Table 3).

**Table 3:** Summary of MHC-IIB variants, genetic divergence and codon-based Z tests for selection in *R. japonica, R. ornativentris,* and *R. t. tagoi*

Species	No. of variants		Range of divergence within individual		Mean distance (nucleotide, amino acid)		Z-tests for selection for exon 2; Z statistic (*p*-value)			Z-tests for selection for exon 3; Z statistic (*p*-value)			
	Total	Per animal	Amino acid	Nucleotide	Amino acid	Exon 2	Exon 3	Neutrality	Purifying	Positive	Neutrality	Purifying	Positive
								d_N_ - d_S_	d_S_ - d_N_	d_N_ - d_S_	d_N_ - d_S_	d_S_ - d_N_	d_N_ - d_S_
*R. japonica*	9	2–3	62	0.001–0.128	0.004–0.233	0.089, 0.179	0.044, 0.063	3.648 (0.0003)	−3.669 (n.s.)	3.641 (0.0002)	−2.543 (0.012)	2.563 (0.006)	−2.573 (n.s.)
*R. ornativentris*	11	1–2	74	0.001–0.158	0–0.262	0.107, 0.180	0.039, 0.044	0.746 (0.457)	−0.743 (n.s.)	0.751 (0.227)	−3.377 (0.001)	3.398 (0.0005)	−3.390 (n.s.)
*R. t. tagoi*	13	2	113	0.011–0.258	0–0.391	0.143, 0.235	0.066, 0.082	0.888 (0.376)	−0.887 (n.s.)	0.881 (0.190)	−3.247 (0.002)	3.334 (0.0006)	−3.262 (n.s.)
*R. t. tagoi *(excl *Rata*-01)			77	0.011–0.153	0–0.234	0.131, 0.220	0.037, 0.036	1.048 (0.297)	−1.049 (n.s.)	1.054 (0.147)	−3.411 (0.001)	3.438 (0.0004)	−3.386 (n.s.)

^#^excluding insertions and deletions; n.s. – not significant, *p* = 1.000

Selection tests over the entire β1 or β2 domains (exons 2 or 3, respectively) indicated that only the β1 domain of *R. japonica* is under balancing selection (Z-value = 3.641, *p* < 0.0001), while the β2 domain of all three focal species is under purifying selection (Table 3). Nevertheless, we identified 22 to 29 codon sites with an excess of non-synonymous substitutions by omegaMap; most sites contain multiple amino acid replacements, which is supportive of balancing selection. The majority of the ‘positive selected sites’ (18–21 sites) were located within exon 2 which encodes the functionally important β1 domain (Fig. 2). Of the 16 important peptide-binding residues in amphibians defined by Bataille et al. [18], 11 sites were also identified as under positive selection in all three Japanese *Rana* species studied. Recombination breakpoints were identified using GARD with significant topological incongruence (*p* < 0.05) in *R. ornativentris* (breakpoint at nucleotide positions 272 and 352) and *R. t. tagoi* (breakpoint at position 344).

### Supertyping analyses

**Fig. 4:**
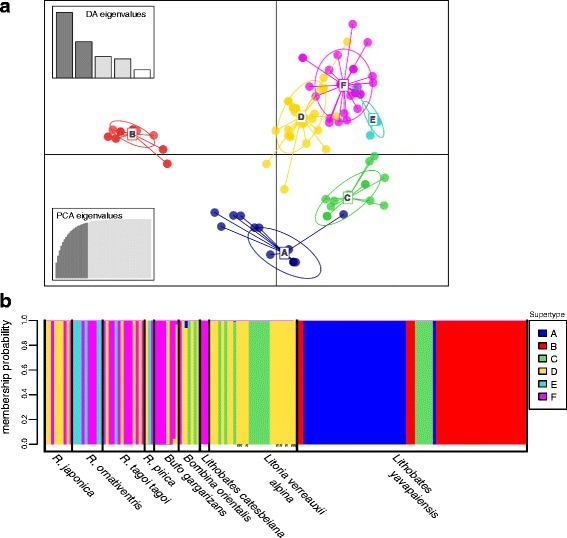
**a** Supertype scatterplot of MHC-IIB alleles or variants (dots) from the three Japanese *Rana* species and other species studied allocated to one of six supertypes (ellipses, ST-A to ST-F); the bottom-left graph represents the cumulative variance retained by 20 principal components, and the top-left graph represents eigenvalues retained for the discriminant analysis. **b** The membership probability of each allele or variant from the nine species used to allocate supertypes (alleles from *L. verreauxii alpina* considered associated with Bd resistance by Bataille et al. [18] are indicated by ‘R’)

MHC-IIB alleles and variants from the three Japanese *Rana* species along with alleles from resistant and susceptible frogs of other studies were assigned to one of six supertypes (ST), ST-A to ST-F, by high membership probability (Fig. 4, Additional file 1: figure S2). Within each of the Japanese *Rana* species, MHC-IIB variants were allocated to at least two supertypes: *R. japonica* in ST-D and -F, *R. ornativentris* to ST-E and -F, and both *R. t. tagoi* and *R. pirica* assigned to ST-D, −E and -F (Additional file 1: Table S8). Variants from resistant South Korean frogs were allocated to ST-C, −D, and -F, while those from resistant carrier *Lithobates catesbeianus* were confined to ST-F. Three supertypes (ST-A to ST–C) from this study were similar to four supertypes (ST1 to ST4) identified in *Lithobates yavapaiensis* by Savage and Zamudio [23]: ST-A equivalent to ST1, ST-B equivalent to ST2 and ST3 which could not be differentiated in our dataset, and ST-C equivalent to ST4. ST-A and -B were specific to *L. yavapaiensis*, while ST-C included two variants from *B. orientalis* (South Korea) and ten variants from *Litoria verreauxii alpina* (Australia). The remaining 19 variants from *L. verreauxii alpina* were allocated to ST-D, and include the eight alleles found in survivors following Bd infection [18].

## Discussion

This study is one of the first transcriptome reports from Japanese frogs and includes three species considered resistant to the deadly worldwide fungal disease caused by *Batrachochytridium dendrobatidis*. We have produced a transcriptome data set focusing on immune genes of healthy frogs, which provides a beneficial framework for future studies. Here, we discuss some applications of this transcriptomic resource and then present one example of such application: an in-depth examination of MHC-IIB, an immune gene potentially important for chytrid fungus resistance.

A comparison of differentially expressed immune genes between adult tissues indicated that immune genes are most highly expressed in the spleen, the major lymphatic tissue in amphibians [29]. Spleen was also found to be more enriched for immune function relative to skin in a transcriptome study of *Lithobates yavapaiensis* [28]. The skin of all three focal species was enriched for the GO term for regulation of inflammatory response. This supports the idea that amphibian skin may be a critical first line of defense against pathogens in the environment. Amphibian skin is a rich source of anti-microbial peptides (or AMPs), which have been shown to have an important role in innate defense against pathogens such as Bd [30]. Each species excretes an unique array of AMPs, and previous studies in all the focal Japanese frogs have isolated a number of AMPs from skin including japonicin-1 and -2, brevinin-1 and -2, palustrin-2, ranatuerin-2, and temporin families [24, 31, 32]. The transcriptomes we have generated in this study have provided an initial overview of the full AMP repertoire of the three species, including several previously described AMP families, and confirmed high expression predominantly in the skin. Our transcriptome data set provides a robust framework for future in-depth analyses of AMP diversity as well as comparative genomics between amphibian species that differ in Bd susceptibility.

Another focus of this study is the characterization of the MHC-IIB gene, due to its potential role in recognizing and binding of Bd-related peptides. This is supported by recent chytridiomycosis-MHCII association studies [18, 22, 23]. We characterized MHC-IIB in the three focal Japanese *Rana* species to explore whether selection has occurred at this gene. In addition, we have compared MHC-IIB expression levels within different adult tissues and with tadpoles. A comparison within adults showed that tissues with enriched MHC class II-related GO terms, which include genes related to peptide binding and MHC class II assembly and transport, also had higher expression levels of MHC-IIB genes. Unlike MHC class I, which is expressed in all nucleated somatic cells, class II is expressed predominantly on antigen-presenting cells such as dendrocytes, macrophages and some lymphocytes. MHC-IIB appears to be most highly expressed in the spleen of the Japanese *Rana*. This aligns with Savage et al. [28] where enrichment of specific GO terms in *L. yavapaiensis* spleen transcriptome suggested a role for MHC-based immunity. However, the limited number of samples here prevents further conclusions (*n* = 3). Indeed, expression of specific MHC-IIB loci could vary across tissues; for example, the *Rata*01 variant has highest expression in blood of the single *R. t. tagoi* transcriptome (Table 2), and this variant also is of interest due to its seemingly divergent phylogenetic history (Fig. 3).

Surprisingly, GO analyses suggested that MHCII-related terms were upregulated in tadpoles of *R. ornativentris* (*n* = 2); this contrasts with transcript expression levels in *R. japonica* and *R. ornativentris* where expression was lowest in tadpoles (Table 2). This was resolved following further examination of the enriched GO terms which indicated that differentially expressed genes were related to transport and assembly of MHC-II proteins rather than the actual molecule. One possibility is that during pre-metamorphosis, MHC class II is immature and the MHC class II-making machinery is up-regulated during the tadpole life stage. Studies in tadpoles of the unrelated *Xenopus* frogs suggest a different pattern whereby MHC class II is ubiquitously expressed during pre-metamorphosis and MHC class I is low until post-metamorphosis [33, 34]. Class I transcripts in the tadpole of *R. ornativentris* were also lower than adults samples, but limited sample size in this study prevents any conclusions for MHC expression during development. To elucidate whether there are true developmental differences in the Japanese *Rana* relative to *Xenopus*, in the future we can investigate MHC class I and II expression levels throughout the tadpole life cycle until metamorphosis. We can utilize the transcriptome resources established in this study to further examine genes important for innate immunity (e.g. complement system, antimicrobial peptides) and adaptive immunity (e.g. MHC class I and II, and genes related to MHC assembly and transport) for quantitative studies of expression level changes and investigation of developmental immunogenetics.

Using primers developed from our transcriptome data, we characterized MHC-IIB from multiple individuals using molecular cloning. Subsequently, we analyzed and identified evidence of the major evolutionary mechanisms that have driven MHC-IIB diversity in the three focal species: balancing selection (e.g. trans-species polymorphism, detection of codon sites with excess of non-synonymous substitutions), recombination and gene duplication. In the three species, signatures of balancing selection were most prominent, especially at the functionally important β1 domain, with identification of many positively selected codon sites in all three species, as well as global excess of non-synonymous substitutions in *R. japonica*. There was also evidence of trans-species polymorphism, with variants across species phylogenetically clustered with high bootstrap support. In particular there were identical MHC-IIB sequences (*Rata*04 and *Raor*09) shared between a *R. t. tagoi* and a *R. ornativentris* individual, despite these two species being more evolutionarily divergent relative to *R. japonica* [35]. Of the other potential mechanisms driving MHC diversity, gene duplication was detected in *R. japonica* only, while recombination was detected in the other two focal species. The limited evidence of gene duplication and recombination at MHC-IIB in these species is in accordance with the variable number of alleles amplified (one to four) and lack of recombination detection in other ranid frogs [36]. The initial characterization of MHC-IIB in Japanese Rana frogs here can drive future diversity studies of populations across Japan.

This is among the first studies to compare MHC-IIB supertypes across a range of Bd-resistant and Bd-susceptible frogs. While direct experimental evidence of the role of MHC in Bd resistance in Japanese ranids is lacking, we can still speculate that immune genes, including MHC-IIB, contribute to the lack of Bd-related demographic declines in Japan and East Asia. Through supertyping analyses, we obtained an overview of the potential physiochemical properties that may contribute to Bd resistance. Three supertypes (ST-D, ST-E and ST-F) were found in ‘resistant’ frogs from Japan, and ST-D also includes alleles with the advantageous P9 binding pocket conformation (i.e., aromatic β37, acidic Aspβ57, Proβ56 and hydrophobic β60 residues) from resistant frogs of Australia and Korea [18]. Although MHC sequences from Japanese ranids and other Bd-resistant frogs [18] share similar P9 pocket conformations, our observation of MHC sequences (ST4, referred as ST-C in this study) from Bd-resistant North Amrerican *Lithobates yavapaiensis* [23] clustering most closely with MHCII-B alleles from Australian *L. verreauxii alpina* frogs that did not survive Bd-infection [18] suggests that protective effects of MHC supertypes may vary across species. This may indicate that distinct Bd-resistant MHC conformations have independently evolved in different populations or continents (America versus Asia-Pacific). Such differences are plausible considering the long evolutionary distance between Japanese and American ranids [37]. In addition, the complex interaction between host-pathogen-environment could drive variation in the protective effect of MHC-IIB, and background differences in MHC structure from neighboring non-peptide binding residues could cause differences in peptide binding.

Future investigations should focus on the impact of amino acid sequence on MHC peptide binding pocket structure [38] and how this influences MHC-Bd peptide binding affinity, using computational [39] or in vitro [40] functional binding assays. Another approach is to investigate the impact of MHC-IIB supertypes on Bd resistance with laboratory challenge studies. In addition, further characterization of other non-MHC genes is important since resistance to diseases like Bd are likely to be polygenic.

## Conclusions

Here we sequenced the transcriptome of three Japanese *Rana* frog species and investigated potential immunogenetic factors contributing to these species’ resistance to the deadly worldwide disease chytridiomycosis. We characterized MHC-IIB for the first time in three species of Japanese ranids, and also explored its expression and molecular evolution. In addition, we expanded on previous studies of functional interpretation of MHC-IIB diversity, by utilizing supertyping analyses to explore the physiochemical properties of MHC-IIB that may be important for recognizing and binding Bd-related antigens and initiating subsequent resistance. By characterizing MHC-IIB in Japanese frogs and exploring its expression and molecular evolution, we have contributed to further understanding complex host-chytridiomycosis dynamics.

## Methods

### Tissue collection and nucleic acid isolation, library construction and sequencing

We collected tissues from three common Ranidae frog species from Japan: the Japanese brown frog (*Rana japonica*), the montane brown frog (*Rana ornativentris*), and Tago’s brown frog (*Rana tagoi tagoi*). To ensure sufficient sequence coverage, species-specific transcriptome data for the three species were each represented by a single adult individual, with additional tadpole samples for two species. A total of 12 samples were used for transcriptome analysis (Table 1, Additional file 1: Table S7): blood, skin and spleen from a single male adult individual of each species (total nine samples), and three tadpole samples from *R. japonica* and *R. ornativentris*. Adult frogs originated from Hiroshima prefecture, Japan, and previously described in [25] (Additional file 1: Table S9); all animals were housed in laboratory conditions for minimum five weeks before euthanasia and exhibited no clinical signs of any disease, thus considered ‘healthy’. Animals were euthanized through immersion in tricaine methanesulfonate (MS222, 0.5–3 g/L water), and samples were collected from the three individuals for immediate RNA extraction. We also included three tadpole samples, euthanized in the same manner, at different larval stages [41]: (i) stage 29 (s29) *R. japonica* whole body, (ii) s29 *R. ornativentris* whole body (both from Hiroshima prefecture, Japan, and stored in RNA*later* solution (Applied Biosystems, Carlsbad, CA, USA) at -20 °C prior to RNA extraction), and (iii) stage 24 (s24) *R. ornativentris* skin (three individuals combined) originating from Yokohama Nature Observation Forest park (Kanagawa prefecture, Japan). We used ISOGEN (Nippon Gene, Tokyo, Japan) to extract total RNA following manufacturer instructions from the 12 samples. Sequencing libraries were created from 1 to 3 μg RNA of each sample using NEBNext® Poly(A) mRNA Magnetic Isolation Module and NEBNext® Ultra™ RNA Library Prep Kit for Illumina® (New England Bio Labs, MA, USA) and used to perform short DNA sequencing (paired-end 100-bp) with a cDNA Illumina Hiseq2000 sequencing system (12 samples in one lane).

For the MHC-IIB genetic characterization study, we euthanized and collected spleen samples from an additional six adult individuals per species and extracted RNA using the same methods, giving a total of *n* = 7 spleen samples per species. PrimeScript™ RT reagent kit (Takara Bio Inc., Otsu, Japan) was used to synthesize first-strand complementary DNA (cDNA). These cDNA samples were previously used to study MHC class I genes [25].

### De novo transcriptome assembly and gene annotation

From the short-read data generated by Illumina HiSeq2000 sequencing, adaptor sequences and low-quality reads were removed, and we then performed de novo short-read transcriptome assembly using default settings in Trinity version 2.2.0 [42]. Three independent reference transcript data sets were created for each of the three focal species, combining all samples from the same species: *R. japonica* (blood, skin, spleen, s29 tadpole body), *R. ornativentris* (blood, skin, spleen, s24 tadpole skin, s29 tadpole body), and *R. t. tagoi* (blood, spleen, skin).

To obtain open reading frames and amino acid sequences, assembly results were further processed using the Transdecoder program within the Trinotate platform [43]. Assembled transcripts were annotated with NCBI-BLAST-2.2.30 with E-values ≤1e-5 against five databases: the Swissprot protein database (http://www.expasy.ch/sprot), human amino acid sequence dataset GRCh38.p5 from Ensembl (http://www.ensembl.org/), the Pfam database (http://pfam.xfam.org), the KO database (http://www.genome.jp/kegg/ko.html), and the GO database (http://www.geneontology.org/). Top hits from BLAST search were regarded as annotations and compiled into annotation reports. We also conducted preliminary identification of antimicrobial peptides (AMPs), using NCBI-BLAST-2.2.30 with E-values ≤1e-5 for tblastn search of 1875 publically available anuran defense peptides collected from the Uniprot database (http://www.uniprot.org/) against our transcriptome data sets.

### Analysis of differential expression (DE)

Differential expression analysis was conducted between samples within each of the species-specific reference transcripts, with a goal to extract (a) all genes and (b) immune-related genes that have tissue- or stage-specific expression. Reads were aligned to their respective reference transcript using RSEM v 1.3.0 [44] to estimate transcript abundance. EdgeR (v3.4), which permits analysis in the absence of biological replicates, was used to extract differentially expressed (DE) transcripts with dispersion parameter of 0.1. Pairwise sample comparisons were then conducted within each of the three species using false discovery rate (FDR) cut-off of 0.001, and transcripts at least 4-fold differentially expressed were extracted. All DE transcripts collected in each of the 12 samples were encoded with functional information from the annotation reports, including Ensembl ID terms. We then performed gene ontology (GO) enrichment analyses using GOseq [45] on significantly DE genes to get a general overview of tissue- or stage-specific expression. From pairwise comparisons, enriched DE genes were collated with GO-slim categories which represent level two GO parent categories. For immune-related genes, collated DE genes with Ensembl gene IDs were submitted to GO analysis implemented in DAVID [46], and enriched non-parental GO terms under the parent GO term ‘immune system process’ or related to interaction with microorganisms were collected using threshold of gene count over five and *p* < 0.05.

### Identification and characterization of MHC-IIB sequences

We scanned the NCBI-BLAST search results for all MHC-IIB sequences independently in the three species. All MHC-IIB transcript sequences that were full coding sequences, spanning from exons one to four, were retained and confirmed by additional search using NCBI-BLAST-2.4.0 against the nucleotide collection (nr/nt) database. To compare MHC-IIB expression between tissue types and between adult and tadpole frogs, we extracted trimmed mean log expression ratio, or TMM-normalized values [47], for MHC-IIB transcripts in each of the 12 samples generated by RSEM v 1.3.0. We also collected TMM-normalized values for MHC class I transcripts and candidate AMP genes identified from prior tblastn search.

From the alignment of MHC-IIB transcript sequences from *R. japonica* (2 sequences), *R. ornativentris* (1 sequence), and *R. t. tagoi* (2 sequences), we designed degenerate primers using Primer3 [48] to amplify partial coding sequence of MHC-IIB. The forward 5’-GMAKYAYHWGCAGMAASATG-3′ and reverse 5’-CACWCCRGCAAYRATAARYA-3′ primers are located in exons 1 and 4, respectively, and amplify all of exons 2 (β1 domain) and 3 (β2 domain). Polymerase chain reaction (PCR) amplification, cloning reactions and sequencing were conducted on spleen cDNA samples (*n* = 7 per species) following Lau et al. [25], except using a lower PCR annealing temperature of 48 °C.

### MHC-IIB sequence analyses, phylogenetics, and selection tests

Criteria for inclusion of MHC-IIB sequences followed Lau et al. [25] to avoid PCR and cloning artifacts, briefly: (1) amplified from more than one clone, and (2) differed to other sequences by more than two nucleotides. Exceptions were allowed for sequences that differed by one or two non-synonymous substitutions, as they were confirmed by repeat PCR and cloning reactions. For phylogenetic analyses, partial MHC-IIB coding sequences from the three focal species were aligned in MEGA7 [49] with that of other amphibians: *Rana pirica* (Mori Tsukasa, unpublished transcriptome data), *L. catesbeianus* [50] (transcriptome data), *Rhinella marina* [51], *Xenopus tropicalis* [52], *X. laevis* [53], *Ambystoma mexicanum* [54], *A. tigrinum* [55], *Quasipaa spinosa* [16], and *Andrias davidianus* [56]. Neighbor joining trees (p-distance, 1000 bootstrap replicates) were constructed from amino acid alignments for the coding sequence of exon 2 (β1 domain) and exon 3 (β2 domain). Maximum likelihood trees (WAG + G model, 1000 bootstrap replicates) were also constructed in MEGA7 to confirm phylogenetic relationships.

Molecular evidence of balancing selection can be inferred by an excess of nonsynonymous (d_N_) to synonymous (d_S_) substitutions, and we tested d_N_ – d_S_ to assess if historical balancing selection has shaped MHC-IIB sequence diversity in Japanese *Rana*. Firstly, we used Z-tests for positive (d_N_ > d_S_), neutral (d_N_ = d_S_) and purifying (d_N_ < d_S_) selection in MEGA7 to assess if selection is acting globally in β1 and β2 domain sequences, for each species independently. We also used a codon-based approach, omegaMap version 5.0 [57], to identify individual codons under selection (d_N_/d_S_ or ω > 1 with a posterior probability of >0.95) even in the presence of recombination. This was conducted for sequence alignments for each of the species independently, and selection parameter (ω) and population recombination rate (ρ) was co-estimated and allowed to vary along the sequence. Two independent runs were conducted following Lau et al. [58] with 1 × 10^6^ Markov chain Monte Carlo iterations, and results were visualized and interpreted using R v 3.3.2 [59]. To test for presence of recombination, we used GARD (Genetic Algorithm Recombination Detection) [60], implemented in the Datamonkey website (http://www.datamonkey.org).

### Supertyping analyses

Supertyping analyses were used to characterize the functional diversity of the MHC-IIB variants identified from the focal Japanese *Rana* frog species compared to that of six other frog species: resistant *Bombina orientalis* and *Bufo gargarizans* from South Korea [18]; resistant carrier *L. catesbeiana* (transcriptome data, DRA accession number SRP051787); resistant *R. pirica* from Hokkaido Japan (Mori, T., unpublished transcriptome data); and *Lithobates yavapaiensis* from North America [23] and *Litoria verreauxii alpina* from Australia [18], both of which are susceptible species with resistant populations or individuals. The supertyping method groups alleles based on similar physiochemical properties at specified amino acid sites. Following Savage and Zamudio [23], we aligned all MHC-IIB amino acid sequences identified from the focal species with that of the other species, and extracted 13 codon positions from the β1 domain (exon 2) identified as peptide-binding region in frogs [18]. Each of the 13 sites were characterized for five physiochemical descriptor variables: z1 (hydrophobicity), z2 (steric bulk), z3 (polarity), z4 and z5 (electronic effects) [61, 62]. Using adegenet 2.0.1 in R v 3.3.2, discriminant analysis of principle components (DAPC) was used to define functional genetic clusters. Using K-means clustering algorithm with Bayesian information criterion (BIC), alleles or variants were classified into clusters that represented distinct MHC supertypes.

## Additional file

Additional file 1: Supplementary information, including **Tables S1–S9**, and **Figures S1–S2**. (DOCX 267 kb)

## Abbreviations

AMPs: antimicrobial peptides
Bd: *Batrachochytridium dendrobatidis*
GO: gene ontology
MHC: major histocompatibility complex
MHC-IIB: major histocompatibility complex class II beta chain
ST: supertype
