# Supplementary material for: Transcriptome analyses of immune tissues from three Japanese frogs (genus Rana) reveals their utility in characterizing major histocompatibility complex class II
Source: BMC Genomics. 2017 Dec 28;18:994. doi: 10.1186/s12864-017-4404-0 (PMC5745589; doi:10.1186/s12864-017-4404-0)

Supplementary Table 1: Summary of differentially expressed genes in pairwise comparisons of samples within each species; blood, skin and spleen are all from adult frogs. The values in parentheses represent the number of level two Gene Ontology (GO) terms significantly enriched in each sample- the list of enriched GO terms are summarised in Supplementary Table 2.

| Pairwise comparison | | *R. japonica* | *R. ornativentris* | *R. t. tagoi* |
| --- | --- | --- | --- | --- |
| Blood x Skin | Blood UP | 1021 (26) | 1118 (17) | 1652 (62) |
|  | Skin UP | 2938 (59) | 2581 (50) | 2864 (53) |
| Blood x Spleen | Blood UP | 531 (23) | 582 (8) | 675 (56) |
|  | Spleen UP | 2371 (49) | 1754 (44) | 1652 (31) |
| Skin x Spleen | Skin UP | 1576 (43) | 1979 (44) | 1976 (50) |
|  | Spleen UP | 1383 (32) | 911 (29) | 1520 (30) |
| s29 tadpole body x Blood | s29 tadpole body UP | 1320 (64) | 6903 (74) | - |
|  | Blood UP | 2855 (46) | 5080 (55) | - |
| s29 tadpole body x Skin | s29 tadpole body UP | 2792 (51) | 6385 (76) | - |
|  | Skin UP | 3087 (32) | 5730 (43) | - |
| s29 tadpole body x Spleen | s29 tadpole body UP | 3340 (49) | 6475 (67) | - |
|  | Spleen UP | 3447 (31) | 5502 (42) | - |
| s24 tadpole skin x Blood | s24 tadpole skin UP | - | 7160 (58) | - |
|  | Blood UP | - | 2817 (40) | - |
| s24 tadpole skin x Skin | s24 tadpole skin UP | - | 5744 (48) | - |
|  | Skin UP | - | 2902 (21) | - |
| s24 tadpole skin x Spleen | s24 tadpole skin UP | - | 6660 (51) | - |
|  | Spleen UP | - | 2931 (20) | - |
| s24 tadpole skin x s29 tadpole body | s24 tadpole skin UP | - | 6750 (66) | - |
|  | s29 tadpole body UP | - | 5989 (74) | - |

Supplementary Table 2. Summary of Gene Ontology (GO) enrichment analysis on differentially expressed transcripts run independently in each of the three species. Values represent proportion (in %) of enriched unigenes categorised into GO-slim categories (equivalent to level two GO categories). Level two GO categories that are enriched in a specific adult tissue (Bl-blood, Sk-skin, Sp-spleen) across all three species, or enriched in tadpoles compared to adult tissues in both *R. japonica* and *R. ornativentris*, are italicized and graphically summarized in Figure 1A.

| GO_term | Category | *R. t. tagoi* | | | *R. japonica* | | | | *R. ornativentris* | | | | |
| --- | --- | --- | --- | --- | --- | --- | --- | --- | --- | --- | --- | --- | --- |
|  |  | Bl | Sk | Sp | Bl | Sk | Sp | S29 tad | Bl | Sk | Sp | S29 tad | S24 tad |
| GO:0000003 | ***BP reproduction*** | 0.0 | ***1.1*** | 0.0 | 0.1 | ***0.7*** | 0.3 | 0.4 | 0.0 | ***0.5*** | 0.2 | 0.0 | 0.2 |
| GO:0000902 | BP cell morphogenesis | 0.0 | 1.8 | 1.2 | 0.0 | 0.7 | 1.1 | 1.8 | 0.0 | 0.5 | 0.6 | 0.2 | 1.2 |
| GO:0002376 | ***BP immune system process*** | 1.1 | 0.0 | ***6.3*** | 0.7 | 0.0 | ***4.4*** | 0.0 | 1.1 | 2.7 | ***4.4*** | 0.0 | 0.0 |
| GO:0003013 | ***BP circulatory system process*** | 0.0 | 0.5 | 0.3 | 0.1 | 0.3 | 0.3 | ***0.7*** | 0.0 | 0.2 | 0.2 | ***0.2*** | ***0.4*** |
| GO:0005975 | BP carbohydrate metabolic process | 0.6 | 0.4 | 0.0 | 0.0 | 0.7 | 0.0 | 0.4 | 0.0 | 0.5 | 0.0 | 0.6 | 1.0 |
| GO:0006091 | ***BP generation of precursor metabolites and energy*** | 0.3 | 0.0 | 0.0 | 0.0 | 0.2 | 0.0 | ***0.5*** | 0.3 | 0.3 | 0.3 | ***0.5*** | ***0.3*** |
| GO:0006259 | BP DNA metabolic process |  |  |  | 0.7 | 0.0 | 0.0 | 0.0 | 0.9 | 0.0 | 0.6 | 0.0 | 0.0 |
| GO:0006397 | BP mRNA processing |  |  |  | 0.9 | 0.3 | 0.3 | 0.0 | 0.7 | 0.3 | 0.4 | 0.4 | 0.0 |
| GO:0006399 | BP tRNA metabolic process |  |  |  |  |  |  |  | 0.1 | 0.0 | 0.0 | 0.1 | 0.1 |
| GO:0006412 | ***BP translation*** | ***1.3*** | 0.0 | 0.0 | ***0.9*** | 0.0 | 0.0 | 0.0 | ***0.9*** | 0.0 | 0.5 | 0.8 | 0.0 |
| GO:0006457 | BP protein folding | 0.3 | 0.0 | 0.0 | 0.0 | 0.1 | 0.0 | 0.1 | 0.2 | 0.0 | 0.0 | 0.2 | 0.1 |
| GO:0006461 | BP protein complex assembly | 0.8 | 0.0 | 0.0 | 0.2 | 0.0 | 0.0 | 0.0 | 0.0 | 0.0 | 0.0 | 0.8 | 0.3 |
| GO:0006520 | BP cellular amino acid metabolic process | 0.3 | 0.5 | 0.0 | 4.8 | 0.0 | 2.2 | 0.0 | 0.0 | 0.2 | 0.0 | 0.3 | 0.4 |
| GO:0006605 | BP protein targeting | 1.2 | 0.0 | 0.0 | 0.0 | 0.4 | 0.0 | 0.5 | 0.8 | 0.0 | 0.5 | 0.6 | 0.0 |
| GO:0006629 | ***BP lipid metabolic process*** | 0.2 | ***1.8*** | 0.0 | 0.0 | ***1.8*** | 0.5 | 1.6 | 0.0 | ***1.7*** | 0.2 | 0.0 | 0.0 |
| GO:0006790 | BP sulfur compound metabolic process | 2.3 | 0.5 | 0.0 | 0.0 | 0.5 | 0.1 | 0.3 | 0.0 | 0.1 | 0.0 | 0.2 | 0.4 |
| GO:0006810 | BP transport | 1.0 | 2.8 | 6.6 | 0.0 | 3.3 | 1.5 | 4.9 | 0.5 | 4.6 | 0.5 | 2.7 | 4.1 |
| GO:0006913 | BP nucleocytoplasmic transport | 1.6 | 0.0 | 0.0 | 0.4 | 0.0 | 0.3 | 0.0 | 0.5 | 0.0 | 0.7 | 0.0 | 0.0 |
| GO:0006914 | BP autophagy |  |  |  | 0.5 | 0.0 | 0.3 | 0.0 | 0.5 | 0.0 | 0.0 | 0.2 | 0.0 |
| GO:0006950 | ***BP response to stress*** | 0.0 | 0.0 | ***6.7*** | 1.4 | 1.8 | ***4.8*** | 2.6 | 3.6 | 2.1 | ***5.3*** | 1.5 | 0.0 |
| GO:0007005 | BP mitochondrion organization |  |  |  | 0.4 | 0.3 | 0.0 | 0.0 | 0.7 | 0.4 | 0.4 | 0.5 | 0.5 |
| GO:0007009 | ***BP plasma membrane organization*** | 0.0 | ***0.4*** | 0.0 | 0.0 | ***0.3*** | 0.1 | 0.3 | 0.0 | ***0.4*** | 0.0 | 0.0 | 0.2 |
| GO:0007010 | ***BP cytoskeleton organization*** | 0.0 | ***1.5*** | 1.0 | 0.0 | ***0.7*** | 0.6 | 1.5 | 0.0 | ***0.7*** | 0.3 | 0.2 | 1.2 |
| GO:0007034 | BP vacuolar transport |  |  |  |  |  |  |  | 0.1 | 0.1 | 0.0 | 0.0 | 0.0 |
| GO:0007049 | BP cell cycle |  |  |  |  |  |  |  | 1.1 | 0.0 | 0.0 | 0.8 | 0.4 |
| GO:0007059 | BP chromosome segregation |  |  |  |  |  |  |  | 0.0 | 0.0 | 0.0 | 0.1 | 0.1 |
| GO:0007067 | BP mitotic nuclear division |  |  |  |  |  |  |  | 0.0 | 0.0 | 0.0 | 0.2 | 0.2 |
| GO:0007049 | BP cell cycle | 1.1 | 0.0 | 0.0 | 0.3 | 0.0 | 0.0 | 0.0 |  |  |  |  |  |
| GO:0007067 | BP mitotic nuclear division | 0.4 | 0.0 | 0.0 |  |  |  |  |  |  |  |  |  |
| GO:0007155 | ***BP cell adhesion*** | 0.0 | 2.3 | ***3.6*** | 0.0 | 1.4 | ***2.6*** | 0.7 | 0.0 | 0.9 | ***2.4*** | 0.0 | 1.4 |
| GO:0007165 | ***BP signal transduction*** | 0.0 | 5.7 | ***9.2*** | 0.0 | 2.5 | ***6.7*** | 0.0 | 0.0 | 2.5 | ***6.8*** | 0.0 | 1.3 |
| GO:0007267 | ***BP cell-cell signaling*** | 0.0 | 1.0 | 1.3 | 0.0 | 0.4 | 0.3 | ***1.1*** | 0.0 | 0.0 | 0.2 | ***0.4*** | ***0.8*** |
| GO:0007568 | BP aging | 0.0 | 0.3 | 0.0 | 0.0 | 0.1 | 0.0 | 0.0 | 0.0 | 0.2 | 0.1 | 0.0 | 0.1 |
| GO:0008150 | BP biological_process | 7.9 | 0.0 | 0.0 | 0.0 | 7.3 | 5.1 | 0.0 | 0.0 | 0.0 | 0.0 | 8.9 | 0.0 |
| GO:0008219 | ***BP cell death*** | 0.0 | 1.2 | ***3.2*** | 0.0 | 1.8 | ***2.2*** | 0.0 | 0.0 | 2.2 | ***2.7*** | 1.1 | 0.4 |
| GO:0008283 | ***BP cell proliferation*** | 0.0 | 2.1 | ***3.3*** | 0.0 | 2.1 | ***2.3*** | 0.7 | 0.0 | 2.3 | ***2.5*** | 0.0 | 1.6 |
| GO:0009056 | ***BP catabolic process*** | ***1.9*** | 0.0 | 0.0 | ***1.6*** | 0.0 | 0.0 | 2.4 | ***2.1*** | 0.0 | 0.0 | 1.7 | 0.5 |
| GO:0009058 | ***BP biosynthetic process*** | ***3.8*** | 0.0 | 0.0 | ***7.2*** | 0.0 | 0.0 | 0.0 | ***6.0*** | 0.0 | 0.0 | 0.0 | 0.0 |
| GO:0009790 | ***BP embryo development*** | 0.0 | ***1.1*** | 0.0 | 0.0 | ***0.8*** | 0.3 | 0.4 | 0.0 | ***0.5*** | 0.2 | 0.0 | 0.7 |
| GO:0016192 | ***BP vesicle-mediated transport*** | 0.0 | 1.0 | ***2.7*** | 0.0 | 0.0 | ***1.8*** | 1.7 | 0.0 | 0.8 | ***2.0*** | 0.6 | 1.0 |
| GO:0019748 | ***BP secondary metabolic process*** | 0.0 | ***0.2*** | 0.0 | 0.0 | ***0.1*** | 0.0 | 0.1 | 0.0 | ***0.2*** | 0.0 | 0.0 | 0.1 |
| GO:0021700 | ***BP developmental maturation*** | 0.0 | ***0.3*** | 0.0 | 0.0 | ***0.2*** | 0.1 | 0.1 | 0.0 | ***0.1*** | 0.1 | 0.0 | 0.2 |
| GO:0022607 | BP cellular component assembly | 1.3 | 0.0 | 0.0 | 0.0 | 0.0 | 0.0 | 2.5 | 0.2 | 0.0 | 0.5 | 1.5 | 2.2 |
| GO:0022618 | BP ribonucleoprotein complex assembly | 0.3 | 0.0 | 0.0 | 0.3 | 0.1 | 0.0 | 0.0 | 0.1 | 0.0 | 0.2 | 0.2 | 0.0 |
| GO:0030154 | BP cell differentiation | 0.0 | 4.1 | 2.7 | 0.0 | 2.7 | 4.1 | 3.8 | 0.0 | 1.8 | 1.4 | 1.4 | 2.6 |
| GO:0030198 | BP extracellular matrix organization | 0.0 | 1.0 | 3.2 | 0.0 | 0.5 | 0.5 | 0.7 | 0.0 | 0.4 | 0.3 | 0.0 | 0.6 |
| GO:0030705 | BP cytoskeleton-dependent intracellular transport |  |  |  | 0.0 | 0.0 | 0.0 | 0.2 | 0.0 | 0.0 | 0.0 | 0.0 | 0.1 |
| GO:0032196 | BP transposition |  |  |  | 0.0 | 0.0 | 0.0 | 0.0 |  |  |  |  |  |
| GO:0034330 | BP cell junction organization | 0.0 | 0.7 | 0.7 | 0.0 | 0.6 | 0.1 | 0.2 | 0.0 | 0.6 | 0.1 | 0.0 | 0.3 |
| GO:0034641 | ***BP cellular nitrogen compound metabolic process*** | ***4.0*** | 0.0 | 0.0 | ***7.9*** | 0.0 | 0.0 | 0.0 | ***7.5*** | 0.0 | 4.1 | 1.9 | 0.3 |
| GO:0034655 | ***BP nucleobase-containing compound catabolic process*** | ***1.0*** | 0.0 | 0.0 | ***0.5*** | 0.0 | 0.0 | 0.0 | ***0.6*** | 0.0 | 0.0 | 0.5 | 0.6 |
| GO:0040007 | BP growth | 0.0 | 0.9 | 0.0 | 0.2 | 0.4 | 0.5 | 0.6 | 0.0 | 0.3 | 0.2 | 0.0 | 0.8 |
| GO:0040011 | ***BP locomotion*** | 0.0 | 2.6 | ***3.6*** | 0.0 | 1.6 | ***2.8*** | 2.0 | 0.0 | 1.1 | ***1.5*** | 0.0 | 1.2 |
| GO:0042254 | BP ribosome biogenesis | 0.4 | 0.6 | 0.0 | 0.2 | 0.1 | 0.0 | 0.0 | 0.3 | 0.0 | 0.2 | 0.3 | 0.1 |
| GO:0042592 | BP homeostatic process | 0.6 | 0.9 | 2.3 | 0.6 | 1.7 | 1.6 | 1.5 | 0.6 | 1.8 | 1.9 | 0.8 | 0.3 |
| GO:0043473 | ***BP pigmentation*** | 0.0 | ***0.2*** | 0.0 | 0.0 | ***0.2*** | 0.0 | 0.0 | 0.0 | ***0.2*** | 0.0 | 0.0 | 0.0 |
| GO:0044281 | ***BP small molecule metabolic process*** | 1.5 | ***3.0*** | 0.0 | 0.0 | ***3.1*** | 0.0 | 3.3 | 0.0 | ***3.4*** | 0.0 | 1.8 | 2.4 |
| GO:0044403 | BP symbiosis, encompassing mutualism through parasitism | 1.3 | 0.0 | 0.9 | 0.8 | 0.0 | 0.8 | 0.0 | 1.0 | 0.0 | 1.0 | 0.8 | 0.0 |
| GO:0048646 | BP anatomical structure formation involved in morphogenesis | 0.0 | 1.4 | 1.0 | 0.0 | 0.9 | 0.7 | 1.3 | 0.0 | 0.4 | 0.5 | 0.0 | 1.1 |
| GO:0048856 | BP anatomical structure development | 0.0 | 5.7 | 6.9 | 0.0 | 3.7 | 5.2 | 5.3 | 0.0 | 2.4 | 1.7 | 1.9 | 4.6 |
| GO:0048870 | ***BP cell motility*** | 0.0 | 1.8 | ***2.7*** | 0.0 | 1.2 | ***2.1*** | 1.0 | 0.0 | 0.8 | ***1.9*** | 0.0 | 1.3 |
| GO:0050877 | ***BP neurological system process*** | 0.0 | 0.8 | 0.0 | 0.0 | 0.5 | 0.0 | ***1.2*** | 0.0 | 0.4 | 0.0 | ***0.5*** | ***0.6*** |
| GO:0051186 | ***BP cofactor metabolic process*** | 0.3 | 0.0 | 0.0 | 0.0 | 0.2 | 0.0 | ***0.4*** | 0.0 | 0.2 | 0.0 | ***0.3*** | ***0.4*** |
| GO:0051276 | BP chromosome organization |  |  |  | 1.8 | 0.0 | 0.0 | 0.0 | 1.2 | 0.0 | 0.0 | 0.2 | 0.0 |
| GO:0051301 | BP cell division | 0.4 | 0.0 | 0.0 | 0.1 | 0.3 | 0.0 | 0.0 | 0.0 | 0.0 | 0.0 | 0.0 | 0.4 |
| GO:0051604 | BP protein maturation | 0.0 | 0.4 | 0.3 | 0.0 | 0.0 | 0.0 | 0.5 | 0.0 | 0.5 | 0.0 | 0.2 | 0.1 |
| GO:0055085 | ***BP transmembrane transport*** | 0.6 | ***1.5*** | 0.0 | 0.0 | ***1.7*** | 0.0 | 1.4 | 0.0 | ***0.7*** | 0.0 | 0.5 | 1.3 |
| GO:0061024 | BP membrane organization | 1.2 | 0.0 | 0.0 |  |  |  |  | 0.0 | 0.7 | 0.0 | 0.9 | 0.3 |
| GO:0065003 | ***BP macromolecular complex assembly*** | ***1.1*** | 0.0 | 0.0 | ***1.5*** | 0.0 | 0.0 | 0.0 | ***1.5*** | 0.0 | 1.0 | 1.1 | 0.4 |
| GO:0071554 | BP cell wall organization or biogenesis | 0.0 | 0.0 | 0.0 | 0.0 | 0.0 | 0.0 | 0.0 | 0.0 | 0.0 | 0.0 | 0.0 | 0.0 |
| GO:0071941 | BP nitrogen cycle metabolic process | 0.0 | 0.0 | 0.0 | 0.0 | 0.0 | 0.0 | 0.0 | 0.0 | 0.0 | 0.0 | 0.0 | 0.0 |
| GO:0000228 | CC nuclear chromosome |  |  |  | 0.6 | 0.0 | 0.0 | 0.0 | 0.0 | 0.0 | 0.0 | 0.3 | 0.0 |
| GO:0000229 | CC cytoplasmic chromosome |  |  |  |  |  |  |  | 0.0 | 0.0 | 0.0 | 0.0 | 0.0 |
| GO:0005575 | ***CC cellular_component*** | 6.0 | ***17.2*** | 0.0 | 0.0 | ***11.7*** | 5.2 | 7.1 | 0.0 | ***11.6*** | 0.0 | 9.1 | 14.5 |
| GO:0005576 | CC extracellular region | 2.3 | 6.5 | 7.2 | 0.0 | 6.3 | 2.5 | 5.8 | 0.0 | 6.4 | 2.8 | 2.7 | 4.3 |
| GO:0005578 | ***CC proteinaceous extracellular matrix*** | 0.0 | ***1.0*** | 0.4 | 0.0 | ***0.5*** | 0.2 | 0.6 | 0.0 | ***0.3*** | 0.2 | 0.1 | 0.6 |
| GO:0005615 | CC extracellular space | 0.0 | 1.9 | 2.7 | 0.0 | 1.9 | 1.4 | 2.2 | 0.0 | 1.8 | 1.5 | 0.6 | 1.1 |
| GO:0005622 | ***CC intracellular*** | ***6.9*** | 0.0 | 0.0 | ***13.1*** | 0.0 | 0.0 | 0.0 | ***12.0*** | 7.2 | 8.2 | 7.7 | 2.9 |
| GO:0005623 | ***CC cell*** | ***7.4*** | 0.0 | 0.0 | ***13.9*** | 0.0 | 0.0 | 0.0 | ***8.3*** | 0.0 | 0.0 | 8.3 | 3.1 |
| GO:0005634 | ***CC nucleus*** | ***3.7*** | 0.0 | 0.0 | ***8.6*** | 0.0 | 0.0 | 0.0 | ***7.8*** | 0.0 | 4.3 | 0.9 | 0.0 |
| GO:0005635 | CC nuclear envelope | 0.4 | 0.0 | 0.0 |  |  |  |  | 0.3 | 0.0 | 0.0 | 0.1 | 0.1 |
| GO:0005654 | CC nucleoplasm |  |  |  | 4.4 | 0.0 | 0.0 | 0.0 | 3.9 | 0.0 | 2.1 | 0.9 | 0.0 |
| GO:0005694 | CC chromosome |  |  |  | 0.8 | 0.0 | 0.0 | 0.0 | 0.6 | 0.0 | 0.0 | 0.5 | 0.0 |
| GO:0005730 | ***CC nucleolus*** | ***2.0*** | 0.0 | 0.0 | ***0.9*** | 0.0 | 0.0 | 0.0 | ***1.1*** | 0.6 | 0.7 | 0.7 | 0.2 |
| GO:0005737 | ***CC cytoplasm*** | 4.4 | 0.0 | 0.0 | 0.0 | 9.5 | 0.0 | ***10.9*** | 5.8 | 5.8 | 6.3 | ***6.3*** | ***9.5*** |
| GO:0005739 | CC mitochondrion |  |  |  | 0.0 | 0.6 | 0.0 | 0.6 | 1.4 | 0.9 | 1.0 | 1.2 | 0.7 |
| GO:0005764 | ***CC lysosome*** | 0.0 | 0.0 | ***0.5*** | 0.0 | 0.5 | ***0.8*** | 0.0 | 0.0 | 0.5 | ***0.5*** | 0.1 | 0.1 |
| GO:0005768 | CC endosome |  |  |  | 0.6 | 0.4 | 1.0 | 0.0 | 0.8 | 0.7 | 1.0 | 0.0 | 0.2 |
| GO:0005773 | ***CC vacuole*** | 0.1 | 0.0 | ***0.6*** | 0.0 | 0.3 | ***0.8*** | 0.0 | 0.0 | 0.4 | ***0.6*** | 0.1 | 0.1 |
| GO:0005777 | CC peroxisome |  |  |  | 0.1 | 0.0 | 0.0 | 0.2 | 0.1 | 0.0 | 0.0 | 0.1 | 0.0 |
| GO:0005783 | ***CC endoplasmic reticulum*** | 0.0 | ***2.1*** | 0.0 | 0.0 | ***2.2*** | 0.5 | 1.9 | 0.0 | ***2.3*** | 0.0 | 0.7 | 1.6 |
| GO:0005794 | ***CC Golgi apparatus*** | 0.0 | ***1.8*** | 0.0 | 0.0 | ***0.4*** | 0.0 | 0.0 | 0.0 | ***2.0*** | 0.0 | 0.0 | 1.4 |
| GO:0005811 | CC lipid particle | 0.2 | 0.0 | 0.0 | 0.0 | 0.1 | 0.0 | 0.0 | 0.0 | 0.0 | 0.0 | 0.0 | 0.0 |
| GO:0005815 | CC microtubule organizing center |  |  |  |  |  |  |  | 0.5 | 0.0 | 0.0 | 0.0 | 0.0 |
| GO:0005829 | ***CC cytosol*** | ***2.7*** | 0.0 | 0.0 | ***2.9*** | 0.0 | 0.0 | 2.4 | ***2.5*** | 0.0 | 2.2 | 2.4 | 0.8 |
| GO:0005840 | CC ribosome | 0.9 | 0.0 | 0.0 | 0.2 | 0.0 | 0.0 | 0.0 | 0.4 | 0.0 | 0.2 | 0.4 | 0.0 |
| GO:0005856 | ***CC cytoskeleton*** | 0.0 | ***1.5*** | 0.0 | 0.0 | ***1.8*** | 0.8 | 3.0 | 0.0 | ***1.2*** | 0.0 | 1.0 | 2.3 |
| GO:0005886 | CC plasma membrane | 0.0 | 5.4 | 7.6 | 0.0 | 5.9 | 5.6 | 4.6 | 0.0 | 2.5 | 3.1 | 0.0 | 3.0 |
| GO:0005929 | CC cilium |  |  |  | 0.0 | 0.0 | 0.0 | 0.7 | 0.0 | 0.0 | 0.0 | 0.0 | 0.3 |
| GO:0016023 | CC cytoplasmic, membrane-bounded vesicle | 0.0 | 1.5 | 1.8 | 0.0 | 1.0 | 1.3 | 1.4 | 0.0 | 1.6 | 0.2 | 0.7 | 1.2 |
| GO:0030312 | CC external encapsulating structure |  |  |  |  |  |  |  | 0.0 | 0.0 | 0.0 | 0.0 | 0.0 |
| GO:0043226 | ***CC organelle*** | ***6.3*** | 0.0 | 0.0 | ***8.8*** | 0.0 | 0.0 | 0.0 | ***10.7*** | 6.7 | 7.5 | 7.2 | 2.7 |
| GO:0043234 | CC protein complex |  |  |  |  |  |  |  | 2.5 | 0.0 | 2.7 | 2.7 | 2.8 |
| GO:0000988 | MF transcription factor activity, protein binding |  |  |  | 0.8 | 0.0 | 0.0 | 0.0 | 0.7 | 0.0 | 0.4 | 0.0 | 0.0 |
| GO:0001071 | MF nucleic acid binding transcription factor activity |  |  |  | 1.3 | 0.0 | 0.0 | 0.0 | 0.4 | 0.0 | 0.0 | 0.0 | 0.0 |
| GO:0003674 | MF molecular_function | 8.4 | 0.0 | 0.0 | 0.0 | 0.0 | 17.5 | 0.0 |  |  |  |  |  |
| GO:0003677 | MF DNA binding |  |  |  | 2.7 | 0.0 | 0.0 | 0.0 | 2.0 | 0.0 | 0.0 | 0.0 | 0.0 |
| GO:0003723 | ***MF RNA binding*** | ***2.0*** | 0.0 | 0.0 | ***2.2*** | 1.0 | 0.0 | 0.0 | ***2.2*** | 1.1 | 1.4 | 1.6 | 0.5 |
| GO:0003729 | ***MF mRNA binding*** | ***0.2*** | 0.0 | 0.0 | ***0.3*** | 0.1 | 0.1 | 0.0 | ***0.2*** | 0.0 | 0.0 | 0.1 | 0.0 |
| GO:0003735 | ***MF structural constituent of ribosome*** | ***0.9*** | 0.0 | 0.0 | ***0.2*** | 0.0 | 0.0 | 0.0 | ***0.3*** | 0.0 | 0.0 | 0.4 | 0.0 |
| GO:0003924 | ***MF GTPase activity*** | 0.2 | 0.0 | 0.0 | 0.0 | 0.0 | 0.0 | ***0.3*** | 0.1 | 0.0 | 0.0 | ***0.2*** | ***0.2*** |
| GO:0004386 | MF helicase activity |  |  |  | 0.2 | 0.0 | 0.0 | 0.0 | 0.2 | 0.0 | 0.0 | 0.0 | 0.0 |
| GO:0004518 | MF nuclease activity |  |  |  | 0.2 | 0.0 | 0.0 | 0.0 | 0.3 | 0.0 | 0.0 | 0.0 | 0.0 |
| GO:0004871 | ***MF signal transducer activity*** | 0.0 | 1.3 | ***2.3*** | 0.0 | 0.6 | ***1.9*** | 0.0 | 0.0 | 0.5 | ***1.7*** | 0.0 | 0.3 |
| GO:0005198 | MF structural molecule activity | 1.1 | 1.1 | 0.7 | 0.0 | 0.8 | 0.3 | 1.5 | 0.0 | 0.5 | 0.2 | 0.9 | 1.0 |
| GO:0008092 | ***MF cytoskeletal protein binding*** | 0.0 | 1.2 | 1.6 | 0.0 | 0.9 | 0.5 | ***1.6*** | 0.0 | 0.6 | 0.4 | ***0.6*** | ***1.1*** |
| GO:0008134 | MF transcription factor binding | 0.0 | 0.0 | 0.5 | 0.7 | 0.0 | 0.4 | 0.0 | 0.7 | 0.0 | 0.0 | 0.0 | 0.0 |
| GO:0008135 | ***MF translation factor activity, RNA binding*** | ***0.2*** | 0.0 | 0.0 | ***0.1*** | 0.0 | 0.0 | 0.0 | ***0.1*** | 0.0 | 0.0 | 0.1 | 0.0 |
| GO:0008168 | MF methyltransferase activity |  |  |  | 0.2 | 0.2 | 0.0 | 0.0 | 0.0 | 0.2 | 0.0 | 0.0 | 0.0 |
| GO:0008233 | ***MF peptidase activity*** | 0.4 | 0.0 | ***1.6*** | 0.0 | 0.0 | ***0.3*** | 1.0 | 0.0 | 0.0 | ***0.2*** | 0.1 | 0.0 |
| GO:0008289 | ***MF lipid binding*** | 0.0 | ***0.8*** | 0.5 | 0.0 | ***0.5*** | 0.3 | 0.9 | 0.0 | ***0.3*** | 0.2 | 0.1 | 0.2 |
| GO:0008565 | MF protein transporter activity | 0.1 | 0.0 | 0.0 |  |  |  |  | 0.1 | 0.0 | 0.0 | 0.1 | 0.0 |
| GO:0016301 | MF kinase activity |  |  |  | 0.0 | 0.0 | 0.2 | 0.0 |  |  |  |  |  |
| GO:0016491 | ***MF oxidoreductase activity*** | 0.0 | ***1.4*** | 0.0 | 0.0 | ***1.4*** | 0.0 | 1.2 | 0.0 | ***1.6*** | 0.6 | 0.7 | 0.8 |
| GO:0016757 | MF transferase activity, transferring glycosyl groups | 0.0 | 0.4 | 0.0 | 0.4 | 0.0 | 0.0 | 0.0 | 0.2 | 0.1 | 0.0 | 0.0 | 0.0 |
| GO:0016765 | MF transferase activity, transferring alkyl or aryl (other than methyl) groups | 0.1 | 0.0 | 0.0 | 0.0 | 0.0 | 0.0 | 0.1 | 0.0 | 0.0 | 0.0 | 0.0 | 0.1 |
| GO:0016779 | MF nucleotidyltransferase activity |  |  |  | 0.1 | 0.0 | 0.0 | 0.0 | 0.2 | 0.0 | 0.0 | 0.1 | 0.0 |
| GO:0016791 | MF phosphatase activity | 0.0 | 0.0 | 0.3 |  |  |  |  |  |  |  |  |  |
| GO:0016798 | MF hydrolase activity, acting on glycosyl bonds | 0.1 | 0.0 | 0.0 | 0.0 | 0.0 | 0.2 | 0.0 |  |  |  |  |  |
| GO:0016810 | MF hydrolase activity, acting on carbon-nitrogen (but not peptide) bonds |  |  |  | 0.0 | 0.0 | 0.0 | 0.2 |  |  |  |  |  |
| GO:0016829 | ***MF lyase activity*** | 0.2 | 0.0 | 0.0 | 0.1 | 0.0 | 0.0 | ***0.3*** | 0.0 | 0.0 | 0.0 | ***0.2*** | ***0.2*** |
| GO:0016853 | MF isomerase activity | 0.1 | 0.0 | 0.0 | 0.0 | 0.1 | 0.0 | 0.1 | 0.0 | 0.1 | 0.0 | 0.1 | 0.1 |
| GO:0016874 | MF ligase activity |  |  |  | 0.5 | 0.0 | 0.0 | 0.0 |  |  |  |  |  |
| GO:0016887 | MF ATPase activity | 0.4 | 0.0 | 0.0 | 0.1 | 0.0 | 0.0 | 0.2 | 0.0 | 0.0 | 0.0 | 0.4 | 0.3 |
| GO:0019843 | ***MF rRNA binding*** | ***0.2*** | 0.0 | 0.0 | ***0.1*** | 0.0 | 0.0 | 0.0 | ***0.1*** | 0.0 | 0.0 | 0.1 | 0.0 |
| GO:0019899 | MF enzyme binding | 1.1 | 0.0 | 0.0 |  |  |  |  | 0.0 | 0.0 | 0.0 | 0.8 | 0.0 |
| GO:0022857 | ***MF transmembrane transporter activity*** | 0.4 | ***1.0*** | 0.0 | 0.0 | ***1.2*** | 0.0 | 1.0 | 0.0 | ***0.5*** | 0.0 | 0.0 | 0.6 |
| GO:0030234 | MF enzyme regulator activity | 0.0 | 1.5 | 1.8 | 0.0 | 1.5 | 1.3 | 1.2 | 0.0 | 1.4 | 1.2 | 0.0 | 0.0 |
| GO:0030674 | ***MF protein binding, bridging*** | 0.0 | ***0.2*** | 0.2 | 0.0 | ***0.2*** | 0.1 | 0.2 | 0.0 | ***0.1*** | 0.1 | 0.0 | 0.1 |
| GO:0032182 | ***MF ubiquitin-like protein binding*** | ***0.1*** | 0.0 | 0.0 | ***0.1*** | 0.0 | 0.0 | 0.0 | ***0.1*** | 0.0 | 0.0 | 0.1 | 0.0 |
| GO:0042393 | MF histone binding |  |  |  | 0.3 | 0.0 | 0.0 | 0.0 | 0.2 | 0.0 | 0.0 | 0.0 | 0.0 |
| GO:0051082 | MF unfolded protein binding | 0.3 | 0.0 | 0.0 |  |  |  |  | 0.0 | 0.0 | 0.0 | 0.1 | 0.0 |

Supplementary Table 3: Immune-related GO terms that are enriched in specific samples within *Rana japonica*.

| Term | Count | PValue |
| --- | --- | --- |
| *Spleen (46 terms)* |  |  |
| GO:0006954~inflammatory response | 150 | 8.44E-12 |
| GO:0050900~leukocyte migration | 60 | 2.61E-09 |
| GO:0045087~innate immune response | 141 | 1.51E-05 |
| GO:0030217~T cell differentiation | 19 | 2.05E-05 |
| GO:0042110~T cell activation | 25 | 2.37E-05 |
| GO:0034162~toll-like receptor 9 signaling pathway | 12 | 6.31E-05 |
| GO:0031295~T cell costimulation | 35 | 7.21E-05 |
| GO:0050728~negative regulation of inflammatory response | 35 | 9.82E-05 |
| GO:0007159~leukocyte cell-cell adhesion | 16 | 3.33E-04 |
| GO:0002224~toll-like receptor signaling pathway | 16 | 3.33E-04 |
| GO:0043029~T cell homeostasis | 16 | 5.61E-04 |
| GO:0050727~regulation of inflammatory response | 28 | 7.22E-04 |
| GO:0042102~positive regulation of T cell proliferation | 27 | 7.46E-04 |
| GO:0050690~regulation of defense response to virus by virus | 15 | 0.002049 |
| GO:0034138~toll-like receptor 3 signaling pathway | 7 | 0.00303 |
| GO:0042130~negative regulation of T cell proliferation | 18 | 0.003227 |
| GO:0006955~immune response | 124 | 0.00349 |
| GO:0002755~MyD88-dependent toll-like receptor signaling pathway | 16 | 0.004613 |
| GO:0050852~T cell receptor signaling pathway | 50 | 0.005787 |
| GO:0045579~positive regulation of B cell differentiation | 9 | 0.006989 |
| GO:0046718~viral entry into host cell | 30 | 0.007036 |
| GO:0046638~positive regulation of alpha-beta T cell differentiation | 7 | 0.007277 |
| GO:0050853~B cell receptor signaling pathway | 22 | 0.008369 |
| GO:0009615~response to virus | 38 | 0.008746 |
| GO:0071345~cellular response to cytokine stimulus | 12 | 0.009301 |
| GO:0045600~positive regulation of fat cell differentiation | 20 | 0.009555 |
| GO:0030854~positive regulation of granulocyte differentiation | 6 | 0.009803 |
| GO:0045060~negative thymic T cell selection | 8 | 0.01009 |
| GO:0051023~regulation of immunoglobulin secretion | 5 | 0.012442 |
| GO:0042113~B cell activation | 13 | 0.013407 |
| GO:0001771~immunological synapse formation | 7 | 0.014587 |
| GO:0002250~adaptive immune response | 47 | 0.014683 |
| GO:0050901~leukocyte tethering or rolling | 8 | 0.017465 |
| GO:0051607~defense response to virus | 52 | 0.017782 |
| GO:0001782~B cell homeostasis | 10 | 0.019582 |
| GO:0002674~negative regulation of acute inflammatory response | 6 | 0.021091 |
| GO:0050862~positive regulation of T cell receptor signaling pathway | 6 | 0.021091 |
| GO:0043304~regulation of mast cell degranulation | 6 | 0.021091 |
| GO:0050707~regulation of cytokine secretion | 7 | 0.025769 |
| GO:0030224~monocyte differentiation | 9 | 0.028589 |
| GO:0048535~lymph node development | 9 | 0.028589 |
| GO:0035754~B cell chemotaxis | 5 | 0.03037 |
| GO:2000107~negative regulation of leukocyte apoptotic process | 5 | 0.03037 |
| GO:0034142~toll-like receptor 4 signaling pathway | 9 | 0.041082 |
| GO:0035666~TRIF-dependent toll-like receptor signaling pathway | 12 | 0.046004 |
| GO:0019886~antigen processing and presentation of exogenous peptide antigen via MHC class II | 30 | 0.049053 |
| *Blood (2 terms)* |  |  |
| GO:0045648~positive regulation of erythrocyte differentiation | 10 | 0.006696 |
| GO:0050690~regulation of defense response to virus by virus | 10 | 0.019977 |
| *Skin(7 terms)* |  |  |
| GO:0050727~regulation of inflammatory response | 25 | 0.008579 |
| GO:0050900~leukocyte migration | 42 | 0.008646 |
| GO:0050728~negative regulation of inflammatory response | 29 | 0.011365 |
| GO:0046718~viral entry into host cell | 28 | 0.02481 |
| GO:0050690~regulation of defense response to virus by virus | 12 | 0.046311 |
| GO:0019886~antigen processing and presentation of exogenous peptide antigen via MHC class II | 36 | 0.001293 |
| *s29 tadpole body (2 terms)* |  |  |
| GO:0030449~regulation of complement activation | 16 | 1.53E-04 |
| GO:0006957~complement activation, alternative pathway | 9 | 9.76E-04 |

Supplementary Table 4: Immune-related GO terms that are enriched in specific samples within *Rana ornativentris*.

| Term | Count | PValue |
| --- | --- | --- |
| *Spleen (19 terms)* |  |  |
| GO:0030217~T cell differentiation | 21 | 9.48E-05 |
| GO:0050900~leukocyte migration | 59 | 2.09E-04 |
| GO:0075733~intracellular transport of virus | 29 | 5.47E-04 |
| GO:0006954~inflammatory response | 151 | 8.10E-04 |
| GO:0050860~negative regulation of T cell receptor signaling pathway | 13 | 0.001195 |
| GO:0050852~T cell receptor signaling pathway | 65 | 0.002305 |
| GO:0045579~positive regulation of B cell differentiation | 11 | 0.002765 |
| GO:0050870~positive regulation of T cell activation | 12 | 0.009988 |
| GO:0009615~response to virus | 48 | 0.010569 |
| GO:0019886~antigen processing and presentation of exogenous peptide antigen via MHC class II | 41 | 0.012551 |
| GO:0050727~regulation of inflammatory response | 29 | 0.024265 |
| GO:0043029~T cell homeostasis | 15 | 0.024344 |
| GO:0007159~leukocyte cell-cell adhesion | 14 | 0.028835 |
| GO:0016064~immunoglobulin mediated immune response | 8 | 0.030184 |
| GO:0045060~negative thymic T cell selection | 8 | 0.030184 |
| GO:0046007~negative regulation of activated T cell proliferation | 7 | 0.034044 |
| GO:2000535~regulation of entry of bacterium into host cell | 5 | 0.038392 |
| GO:0050728~negative regulation of inflammatory response | 34 | 0.04026 |
| GO:0051607~defense response to virus | 64 | 0.049444 |
| *Blood (5 terms)* |  |  |
| GO:0075733~intracellular transport of virus | 29 | 4.25E-05 |
| GO:0046718~viral entry into host cell | 35 | 0.00336 |
| GO:0050852~T cell receptor signaling pathway | 57 | 0.005138 |
| GO:0050727~regulation of inflammatory response | 26 | 0.027905 |
| GO:0045059~positive thymic T cell selection | 6 | 0.042766 |
| *Skin (7 terms)* |  |  |
| GO:0046718~viral entry into host cell | 45 | 1.00E-04 |
| GO:0045579~positive regulation of B cell differentiation | 11 | 0.004907 |
| GO:0050860~negative regulation of T cell receptor signaling pathway | 12 | 0.009756 |
| GO:0050727~regulation of inflammatory response | 31 | 0.018302 |
| GO:0075733~intracellular transport of virus | 26 | 0.019808 |
| GO:0019886~antigen processing and presentation of exogenous peptide antigen via MHC class II | 42 | 0.024061 |
| GO:2000535~regulation of entry of bacterium into host cell | 5 | 0.049354 |
| *s29 tadpole body (3 terms)* |  |  |
| GO:0075733~intracellular transport of virus | 35 | 3.08E-05 |
| GO:0002479~antigen processing and presentation of exogenous peptide antigen via MHC class I, TAP-dependent | 37 | 0.001554 |
| GO:0019886~antigen processing and presentation of exogenous peptide antigen via MHC class II | 47 | 0.012412 |
| *s24 tadpole skin (3 terms)* |  |  |
| GO:0075733~intracellular transport of virus | 34 | 0.012794 |
| GO:0050900~leukocyte migration | 70 | 0.038607 |
| GO:0019886~antigen processing and presentation of exogenous peptide antigen via MHC class II | 54 | 0.043622 |

Supplementary Table 5: Immune-related GO terms that are enriched in specific samples within *Rana tagoi tagoi*.

| Term | Count | PValue |
| --- | --- | --- |
| *Spleen (38 terms)* |  |  |
| GO:0030217~T cell differentiation | 19 | 8.31E-10 |
| GO:0006954~inflammatory response | 85 | 8.22E-08 |
| GO:0045087~innate immune response | 89 | 1.35E-06 |
| GO:0050900~leukocyte migration | 35 | 3.70E-06 |
| GO:0050853~B cell receptor signaling pathway | 20 | 1.28E-05 |
| GO:0042110~T cell activation | 18 | 1.73E-05 |
| GO:0031295~T cell costimulation | 23 | 1.33E-04 |
| GO:0002250~adaptive immune response | 35 | 1.49E-04 |
| GO:0007159~leukocyte cell-cell adhesion | 12 | 1.95E-04 |
| GO:0097028~dendritic cell differentiation | 8 | 1.95E-04 |
| GO:0001771~immunological synapse formation | 7 | 4.76E-04 |
| GO:0006955~immune response | 76 | 6.12E-04 |
| GO:0042102~positive regulation of T cell proliferation | 18 | 8.64E-04 |
| GO:0050776~regulation of immune response | 35 | 8.99E-04 |
| GO:0042113~B cell activation | 11 | 9.30E-04 |
| GO:0046718~viral entry into host cell | 21 | 0.001414 |
| GO:0030890~positive regulation of B cell proliferation | 13 | 0.001932 |
| GO:0050690~regulation of defense response to virus by virus | 10 | 0.005046 |
| GO:0043305~negative regulation of mast cell degranulation | 5 | 0.005965 |
| GO:0030889~negative regulation of B cell proliferation | 7 | 0.009394 |
| GO:0030595~leukocyte chemotaxis | 6 | 0.010715 |
| GO:0042100~B cell proliferation | 10 | 0.013004 |
| GO:0030593~neutrophil chemotaxis | 16 | 0.013234 |
| GO:0002224~toll-like receptor signaling pathway | 9 | 0.013634 |
| GO:0042130~negative regulation of T cell proliferation | 11 | 0.01468 |
| GO:0050728~negative regulation of inflammatory response | 18 | 0.014976 |
| GO:0006957~complement activation, alternative pathway | 6 | 0.015666 |
| GO:0034142~toll-like receptor 4 signaling pathway | 7 | 0.017535 |
| GO:0045579~positive regulation of B cell differentiation | 6 | 0.021936 |
| GO:0001782~B cell homeostasis | 7 | 0.02301 |
| GO:0050852~T cell receptor signaling pathway | 28 | 0.025869 |
| GO:0046007~negative regulation of activated T cell proliferation | 5 | 0.026368 |
| GO:0033089~positive regulation of T cell differentiation in thymus | 5 | 0.026368 |
| GO:0046641~positive regulation of alpha-beta T cell proliferation | 5 | 0.026368 |
| GO:0030183~B cell differentiation | 15 | 0.028854 |
| GO:0002755~MyD88-dependent toll-like receptor signaling pathway | 9 | 0.043788 |
| GO:0050727~regulation of inflammatory response | 14 | 0.047046 |
| GO:0043029~T cell homeostasis | 8 | 0.04963 |
| *Blood (6 terms)* |  |  |
| GO:0075733~intracellular transport of virus | 14 | 6.11E-04 |
| GO:0019886~antigen processing and presentation of exogenous peptide antigen via MHC class II | 20 | 7.05E-04 |
| GO:0051607~defense response to virus | 28 | 0.002684 |
| GO:0002474~antigen processing and presentation of peptide antigen via MHC class I | 9 | 0.007619 |
| GO:0043029~T cell homeostasis | 8 | 0.012297 |
| GO:0050727~regulation of inflammatory response | 12 | 0.03286 |
| *Skin (3 terms)* |  |  |
| GO:0046597~negative regulation of viral entry into host cell | 8 | 0.022197 |
| GO:0046718~viral entry into host cell | 21 | 0.023746 |
| GO:0050727~regulation of inflammatory response | 17 | 0.040222 |

Supplementary Table 6: Specific MHC class II –related genes in *R. ornativentris* tadpole samples, within the enriched gene ontology term GO:0019886 (antigen processing and presentation of exogenous peptide antigen via MHC class II)

| Gene | *R. ornativentris* s29 tadpole body | *R. ornativentris* s24 tadpole skin |
| --- | --- | --- |
| Alpha-centractin | ENSG00000138107 | ENSG00000138107 |
| AP-1 and -2 complex | ENSG00000006125, ENSG00000100280, ENSG00000166747, ENSG00000072958, ENSG00000106367, ENSG00000182287, ENSG00000152056, ENSG00000183020, ENSG00000161203 | ENSG00000006125, ENSG00000100280, ENSG00000166747, ENSG00000072958, ENSG00000129354, ENSG00000182287, ENSG00000152056, ENSG00000196961, ENSG00000183020 |
| Aspartic protease 6 | ENSG00000196188 | - |
| Calnexin, Calmegin | ENSG00000127022 | ENSG00000127022 |
| Cathepsin | ENSG00000117984, ENSG00000174080, ENSG00000163131, ENSG00000135047, ENSG00000163131 | ENSG00000117984, ENSG00000136943, ENSG00000163131 |
| Centromere-associated protein | - | ENSG00000138778 |
| Clathrin | ENSG00000122705, ENSG00000141367 | ENSG00000122705, ENSG00000141367 |
| Cysteine proteinase | ENSG00000163131 |  |
| Dynein | ENSG00000077380, ENSG00000197102, ENSG00000187240, ENSG00000264364 | ENSG00000077380, ENSG00000135720, ENSG00000197102, ENSG00000187240 |
| Dynactin | ENSG00000175203, ENSG00000137100, ENSG00000166847, ENSG00000104671, | ENSG00000204843, ENSG00000137100, ENSG00000132912, ENSG00000166847 |
| Dynamin | - | ENSG00000079805 |
| Kinesin | ENSG00000138160, ENSG00000068796, ENSG00000142945, ENSG00000131437, ENSG00000084731, ENSG00000090889, ENSG00000155980, ENSG00000126214 | ENSG00000121621, ENSG00000066735, ENSG00000138160, ENSG00000079616, ENSG00000137807, ENSG00000068796, ENSG00000142945, ENSG00000131437, ENSG00000084731, ENSG00000090889, ENSG00000155980, ENSG00000075945, ENSG00000126214 |
| Legumain | ENSG00000100600 | ENSG00000100600 |
| Oryzain | ENSG00000163131 | - |
| Oxysterol-binding protein-related protein | ENSG00000141447 | - |
| Pepsin | ENSG00000196188 | - |
| Rac GTPase-activating protein | ENSG00000161800 | ENSG00000161800 |
| GTP-binding protein SAR1 | ENSG00000152700 | - |
| Protein transport protein | ENSG00000138802, ENSG00000176986, ENSG00000150961, ENSG00000138674, ENSG00000157020 | ENSG00000100934, ENSG00000113615, ENSG00000176986, ENSG00000150961, ENSG00000138674 |
| Endophilin | ENSG00000107295 | ENSG00000107295 |
| Vacuolar protein sorting-associated protein | ENSG00000079805 | - |
| Xylem cysteine proteinase | ENSG00000136943 | - |
| Ras-related protein | ENSG00000075785 | - |
| TNF receptor-associated factor 6 | - | ENSG00000175104 |

Supplementary Table 7. Normalized expression of all antimicrobial peptide sequences identified from transcriptome data of three Japanese *Rana* species, represented by TMM (trimmed mean of log expression ratio) values.

| Species | Species and AMP | Blood TMM | Skin TMM | Spleen TMM | S24 tadpole skin TMM | S29 tadpole body TMM |
| --- | --- | --- | --- | --- | --- | --- |
| *R. japonica* | Bradykinin-2  Brevinin-1TOa  Brevinin-2LT2  Histone H2B  Japonicin-2Ja  Japonicin-1NPb  Kassorin-M  Kininogen-4  Odorranain-F2  Pleurain-A2  Preprobrevinin-1Ja  Preprobrevinin-2CE  Preprochensinin-1K  Ranacyclin Ca  Ranatensin  Temporin-1P | 0.0  0.0  0.0  3.3  0.0  0.0  0.0  0.0  0.0  0.0  0.0  0.0  0.6  0.0  0.0  0.3 | 0.3  0.7  0.0  3.1  2573.5  22.5  1668.7  145.0  1287.9  143.2  327.0  17.5  582.5  2.7  0.0  815.8 | 0.5  0.0  1.3  1.1  1.9  0.2  0.0  0.0  0.6  0.0  0.2  0.0  1.3  0.0  0.0  0.7 | -  -  -  -  -  -  -  -  -  -  -  -  -  -  -  - | 0.0  26.4  0.0  2.8  0.3  0.0  0.5  0.0  0.0  0.0  62.4  0.0  8.7  0.0  1.6  1.8 |
| *R. ornativentris* | Andersonin-R  Bombesin  Bradykinin-3  Brevinin-1RTb  Gaegurin-6-RN  Histone H2B  Japonicin-2Ja  Kininogen-4  Nigroain-A  Odorranain-V2  Odorranain B5  Peptide DK25  Pleurain-A4  Pleurain-P  Preprobrevinin-1Ka  Preprobrevinin-2Oc  Preprochensinin-2CE  Preprochensinin-1CEa  Prepropalustrin-2Oa  Preproranatuerin-2Oe  Ranacyclin_Ca  Ranatuerin_2C-RA1  Ranatuerin-2YJ  Tigerinin-RC1 | 0.0  0.0  0.0  0.5  1.0  1.9  0.0  0.0  0.0  0.1  0.0  0.0  0.0  0.0  0.0  0.0  0.0  0.6  0.0  0.0  0.0  0.0  0.0  0.0 | 0.0  0.0  0.0  760.2  0.0  1.7  0.0  0.0  0.0  787.4  0.0  0.0  768.4  0.0  572.3  18.3  30.6  514.2  0.7  740.4  1.2  2.4  473.4  0.0 | 0.0  0.0  0.0  1.0  0.0  2.0  0.0  0.0  0.0  0.0  0.0  0.0  0.0  0.0  0.0  0.0  0.0  0.3  0.0  0.0  0.0  0.0  0.0  0.0 | 0.0  0.0  0.7  0.0  0.0  6.5  1.2  0.9  210.1  0.0  175.0  0.8  0.0  0.4  0.0  0.1  0.0  0.0  0.0  0.0  0.0  0.0  0.0  1294.1 | 30.7  8.1  0.0  0.0  0.0  9.9  0.0  0.0  0.0  0.0  0.0  0.0  0.0  1.3  0.0  0.0  0.0  0.0  0.0  0.0  9.3  0.0  0.0  0.0 |
| *R. t. tagoi* | Andersonin-R  Bradykinin-2  Bradykinin-3  Brevinin-1TOa  Daunchinain-D1  Esculentin-2PLa  Gaegurin-6-RN  Histone H2B  Japonicin-1NPb  Kininogen-2  Preprobrevinin-1Ka  Preprobrevinin-2Oc  Preprochensinin-2CE  Prepropalustrin-2Ka  Ranatuerin-2AMb  Temporin-1P_2  Temporin-GN2 | 0.0  0.0  0.0  0.0  0.7  0.0  0.0  0.1  0.0  0.0  0.0  0.0  0.0  0.0  0.0  0.0  0.0 | 20.3  161.5  381.7  222.0  12.0  3.0  989.8  9.2  325.4  87.3  883.1  1.7  28.1  51.9  2.5  0.5  50.2 | 0.0  0.0  0.0  0.0  0.0  0.0  0.0  0.7  0.3  0.0  0.0  0.0  0.0  0.0  0.0  0.2  0.0 | -  -  -  -  -  -  -  -  -  -  -  -  -  -  -  -  - | -  -  -  -  -  -  -  -  -  -  -  -  -  -  -  -  - |

Supplementary Table 8: MHC-IIB variants from multiple frog species were allocated to one of six supertypes (ST-A to ST-F). ^#^Supertypes were similar to those allocated by Savage and Zamudio (2016), e.g. ST1 is equivalent to ST-A from this study, while ST2 and ST3 could not be separated in this study and allocated to ST-B.

| Species | ST-A (ST1^#^) | ST-B (ST2/ST3^#^) | ST-C (ST4^#^) | ST-D | ST-E | ST-F |
| --- | --- | --- | --- | --- | --- | --- |
| Japanese *Rana* (This study) |  |  |  | Raja01, Raja02,  Raja04, Raja05,  Raja06, Raja08,  Rata01, Rata06,  Rata10, Rata12 | Raor01, Raor02,  Raor03, Raor05,  Raor09, Raor10  Rata04 | Raja03, Raja07, Raja09  Raor04, Raor06,Raor07, Raor08,Raor11  Rata02, Rata03, Rata05, Rata07,Rata08, Rata09,  Rata11, Rata13 |
| Rana pirica |  |  |  | Rapi_tr_1 | Rapi_tr_2 | Rapi_tr_3 |
| *Litoria verreauxii alpina* (* indicates ‘resistant’ alleles) |  |  | KJ679297.1_Livea-6  KJ679298.1_Livea-7a  KJ679299.1_Livea-7b  KJ679300.1_Livea-8a  KJ679301.1_Livea-8b  KJ679302.1_Livea-9  KJ679303.1_Livea-10  KJ679308.1_Livea-15  KJ679311.1_Livea-18  KJ679313.1_Livea-20 | KJ679288.1_Livea-1*, KJ679289.1_Livea-2*,  KJ679290.1_Livea-3a, KJ679291.1_Livea-3b*,  KJ679292.1_Livea-4, KJ679293.1_Livea-5a*,  KJ679294.1_Livea-5b*, KJ679295.1_Livea-5c,  KJ679296.1_Livea-5d, KJ679304.1_Livea-11*,  KJ679305.1_Livea-12, KJ679306.1_Livea-13*,  KJ679307.1_Livea-14*, KJ679309.1_Livea-16,  KJ679310.1_Livea-17, KJ679312.1_Livea-19,  KJ679314.1_Livea-21, KJ679315.1_Livea-22,  KJ679316.1_Livea-23 |  |  |
| Korean frogs |  |  | KJ679326.1_Boor-2  KJ679329.1_Boor-5 | KJ679317.1_Buga-1  KJ679320.1_Buga-4  KJ679325.1_Boor-1  KJ679327.1_Boor-3  KJ679330.1_Boor-6  KJ679331.1_Boor-7 |  | KJ679318.1_Buga-2  KJ679319.1_Buga-3  KJ679321.1_Buga-5  KJ679322.1_Buga-6  KJ679323.1_Buga-7  KJ679324.1_Buga-8  KJ679328.1_Boor-4 |
| *Lithobates catesbeianus* |  |  |  |  |  | Lica_BT081564.1  Lica_tr_1  Lica_tr_2 |
| *Lithobates yavapaiensis* | ANQ37091.1, ANQ37101.1,  ANQ37102.1, ANQ37103.1,  ANQ37104.1, ANQ37105.1,  ANQ37106.1, ANQ37107.1,  ANQ37108.1, ANQ37109.1,  ANQ37110.1, ANQ37111.1,  ANQ37112.1, ANQ37113.1,  ANQ37114.1, ANQ37115.1,  ANQ37116.1, ANQ37117.1,  ANQ37118.1, ANQ37119.1,  ANQ37120.1, ANQ37121.1,  ANQ37122.1, ANQ37123.1,  ANQ37124.1, ANQ37125.1,  ANQ37126.1, ANQ37127.1,  ANQ37128.1, ANQ37129.1,  ANQ37130.1, ANQ37131.1,  ANQ37132.1, ANQ37133.1,  ANQ37134.1 | ANQ37059.1, ANQ37060.1, ANQ37061.1, ANQ37062.1,  ANQ37063.1, ANQ37064.1,  ANQ37065.1, ANQ37066.1,  ANQ37067.1, ANQ37069.1,  ANQ37070.1, ANQ37071.1,  ANQ37072.1, ANQ37073.1,  ANQ37074.1, ANQ37075.1,  ANQ37076.1, ANQ37078.1,  ANQ37079.1, ANQ37080.1,  ANQ37081.1, ANQ37082.1,  ANQ37083.1, ANQ37084.1,  ANQ37085.1, ANQ37086.1,  ANQ37087.1, ANQ37088.1,  ANQ37089.1, ANQ37090.1,  ANQ37098.1, ANQ37099.1,  ANQ37100.1, ANQ37135.1,  ANQ37136.1 | ANQ37092.1  ANQ37093.1  ANQ37094.1  ANQ37095.1  ANQ37096.1  ANQ37097.1 |  |  |  |

Supplementary Table 9: Detailed summary of samples used for tissue collection and transcriptome library construction. E– raised in captivity from eggs, T- raised in captivity from tadpoles, A- adults collected in field and held in captivity.

| Species | Life stage (sample/s) | Source | Captivity information | RNA extraction after sample collection? |
| --- | --- | --- | --- | --- |
| *R. japonica* | Adult (blood, skin, spleen)  s29 tadpole (body) | Etajima 34°16'14''N 132°28'37''E  “ “ | E  E | Yes  No (RNAlater) |
| *R. ornativentris* | Adult (blood, skin, spleen)  s29 tadpole (body)  s24 tadpole (skin) | Yoshiwa 34°25'04’’N 132°05'15''E  “ “  Yokohama 35°20'21"N 139°35'09"E | T  T  E | Yes  Yes  No (RNAlater) |
| *R. t. tagoi* | Adult (blood, skin, spleen) | Shobara 34°05'04''N 132°49'43''E | A | Yes |

Supplementary Figure 1: Phylogenetic relationships between MHC-IIB variants (amino acid sequences) identified in *R. japonica (Raja*, orange bar*), R. ornativentris (Raor*, grey bar*), and R. t. tagoi (Rata*, brown bar*)* and other amphibians using maximum likelihood method and 500 bootstrap replicates. Phylogenies were constructed based from (a) entire β1 domain encoded by exon 2, and (c) entire β2 domain encoded by exon 3. Results are very similar to the neighbour joining method in Figure 3.


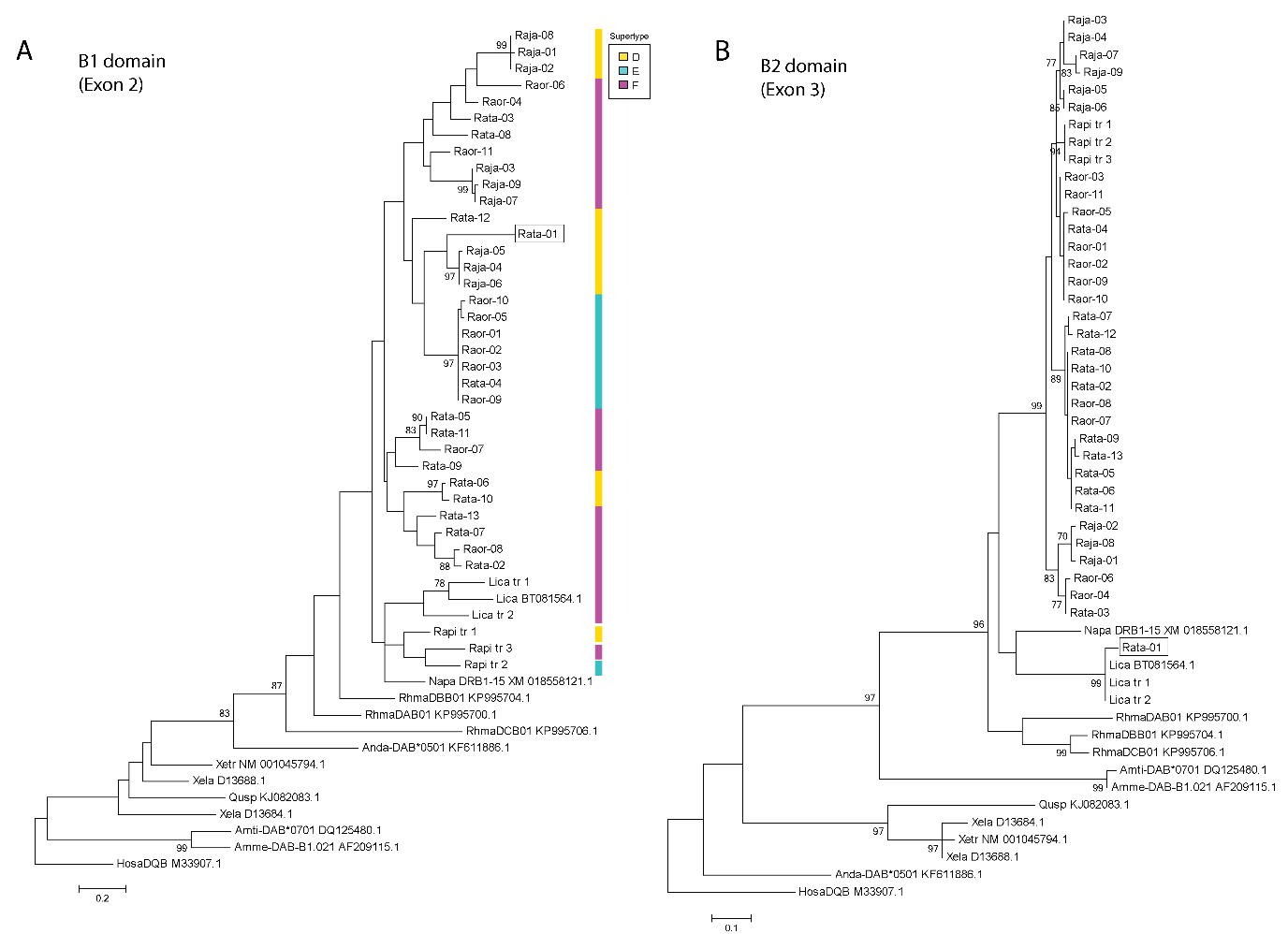


Supplementary Figure 2: Average Z-values for each of the 13 codon sites (peptide binding residues, marked in Figure 1) used for determining supertypes: z1 (hydrophobicity), z2 (steric bulk), z3 (polarity), z4 and z5 (electronic effects). Ten specific z-values contributed statistically to the supertypes determined, as shown in blue boxes.


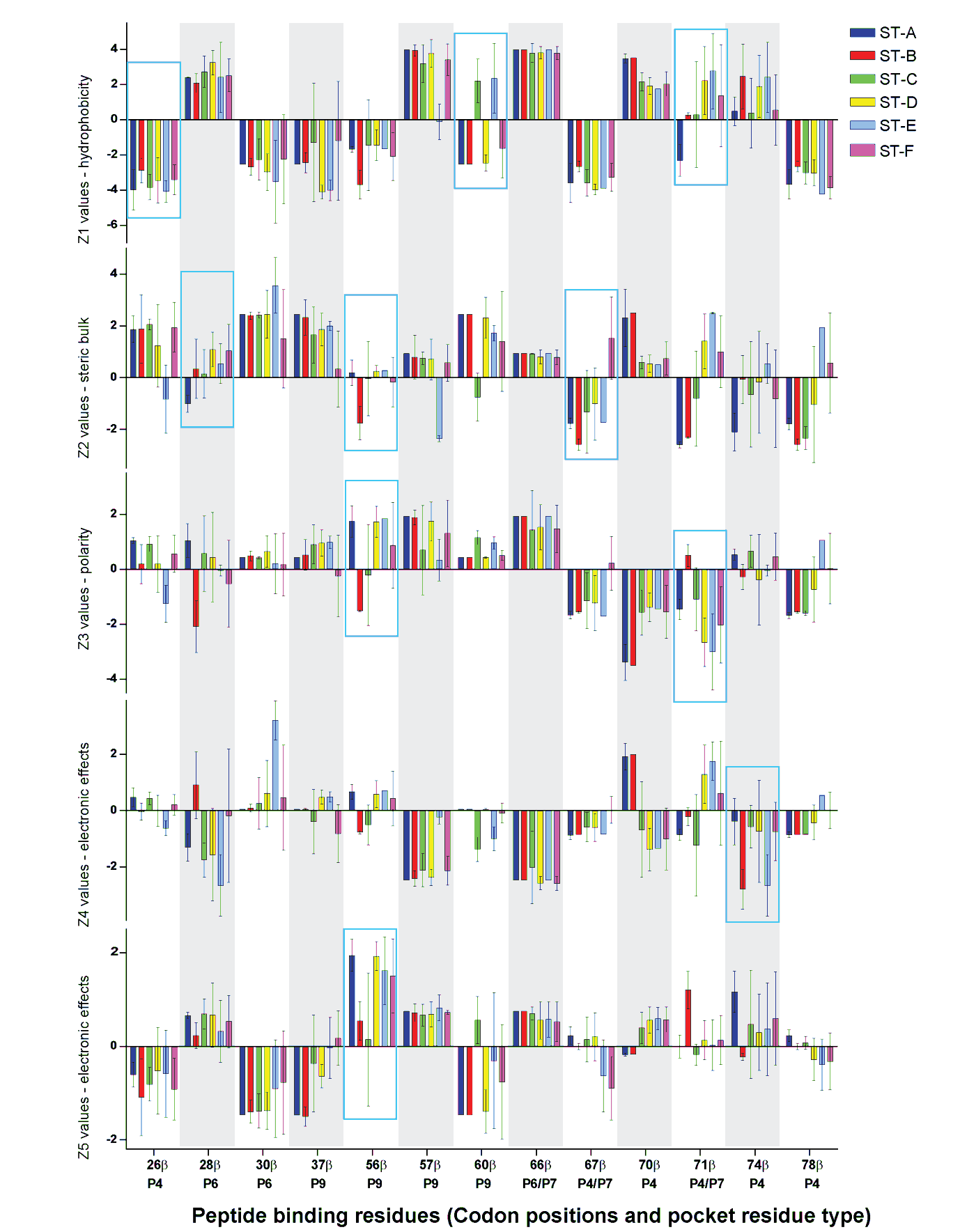

Supplement: Additional file 1: — Supplementary information, including Tables S1–S9, and Figures S1–S2. (DOCX 267 kb) [file 12864_2017_4404_MOESM1_ESM.docx]
